# Analysis of plasma metabolic profile, characteristics and enzymes in the progression from chronic hepatitis B to hepatocellular carcinoma

**DOI:** 10.18632/aging.103554

**Published:** 2020-07-23

**Authors:** Fei-Fei Cai, Ya-Nan Song, Yi-Yu Lu, Yongyu Zhang, Yi-Yang Hu, Shi-Bing Su

**Affiliations:** 1 Research Center for Traditional Chinese Medicine Complexity System, Institute of Interdisciplinary Integrative Medicine Research, Shanghai University of Traditional Chinese Medicine, Shanghai 201203, China; 2 Shanghai Seventh People's Hospital, Shanghai University of Traditional Chinese Medicine, Shanghai 200137, China; 3 Research Center for Traditional Chinese Medicine and System Biology, Institute of Interdisciplinary Integrative Medicine Research, Shanghai University of Traditional Chinese Medicine, Shanghai 201203, China; 4 Institute of Liver Diseases, Shuguang Hospital, Shanghai University of Traditional Chinese Medicine, Shanghai 200203, China

**Keywords:** hepatocellular carcinoma, chronic hepatitis B, liver cirrhosis, metabolite, diagnosis and prognosis

## Abstract

Hepatitis B virus (HBV) infection is an important factor causing hepatocellular carcinoma (HCC). The aim of this study was to investigate the metabolic characteristics and related metabolic enzyme changes during the progression from chronic hepatitis B (CHB) to liver cirrhosis (LC) and, ultimately, to HCC. An untargeted metabolomics assay was performed in plasma from 50 healthy volunteers, 43 CHB patients, 67 LC patients, and 39 HCC patients. A total of 24 differential metabolites (DMs) were identified. Joint pathway analysis suggested striking changes in amino acid metabolism and lipid metabolism from CHB to HCC. The panel of L-serine, creatine and glycine distinguished LC from CHB, and L-serine, cystathionine, creatine and linoleic acid distinguished HCC from LC. Bioinformatic analysis of publicly available data showed that differential metabolite profile-associated enzyme genes, including alanine-glyoxylate aminotransferase-2 (*AGXT2*), D-amino-acid oxidase (*DAO*), and cystathionine gamma-lyase (*CTH*), were downregulated, while bisphosphoglycerate mutase (*BPGM*), cystathionine-β-synthase (*CBS*), phosphoserine phosphatase (*PSPH*) and acyl-CoA thioesterase 7 (*ACOT7*) were upregulated, in HCC, all of which correlated with a poor prognosis for HCC patients. Our results indicated that serum metabolites and related enzymes are of considerable significance for the diagnosis and prognosis of HCC and can provide a theoretical basis and therapeutic index for future diagnosis and treatment.

## INTRODUCTION

It is estimated that one out of three people worldwide have been infected with hepatitis B virus (HBV), and approximately 240 million to 350 million of them will progress to chronic hepatitis [1]. People have been able to effectively prevent HBV infection since the introduction of the hepatitis B vaccine in 1982 [2], however, approximately 1 million people that die from hepatitis B-related chronic liver disease each year [3]. Most patients with chronic hepatitis have no obvious symptoms but have the opportunity to develop cirrhosis or even hepatocellular carcinoma (HCC) [4].

Liver cirrhosis (LC) is a long-term pathological process, and one of the main causes of LC is chronic hepatitis B (CHB). Although alcoholism is estimated to cause 60-70% of cirrhosis, most cirrhosis is caused by viral hepatitis, and HCC is secondary to cirrhosis [5]. HCC is the most common type of chronic liver cancer in adults and is the most common cause of death in patients with liver cirrhosis [6]. In particular, chronic HBV infection can attack hepatocytes by repeatedly inducing the autoimmune system, with some of the hepatocytes being infected by viruses, and chronic HBV infection can contribute to the development of HCC [7]. Therefore, clarifying the mechanism of CHB and cirrhosis development in HCC may help to prevent the development and progression of HCC.

The liver is the most important metabolic organ in the body. The liver participates in almost all metabolic and physiological processes, including metabolism of carbohydrates, proteins and amino acids (including enzymes), lipids, vitamins, hormones, biliary pigments, and bile acids, secretion and excretion of metabolites, and biotransformation of drugs or toxins [8]. The study of metabolic activity can help to characterize the physiological function of the liver and the pharmacological mechanism of drugs for treating liver diseases.

Metabolomics technologies have the methodological characteristic of a holistic view. These technologies regard the research object as a whole and obtain the metabolite information of the research object in an all-round way to determine the changing law of the whole organism [9]. Previous studies have identified the serum metabolic profiles in the progression of HBV infection to HCC using a non-targeted gas chromatography-time of flight-mass spectrometry (GC-TOFMS), and 15 metabolites were determined to be intimately associated with the process [10]. Wu T et al evaluated the dynamic change in serum lipid metabolism during the progression from CHB to HCC by targeted metabolomics analysis and concluded that the serum levels of long-chain lysophosphatidylcholines (lysoPCs) were promising markers of HBV-associated carcinoma [11]. In addition, most of the related studies on HCC metabolism have focused on glucose metabolism [12, 13] and fatty acids metabolism [14], and relatively little is known about other metabolic pathways.

In the present study, we used gas chromatography mass spectrometry (GC/MS) to explore the dynamic changes in plasma metabolic profiles, characteristic metabolites and metabolic pathways in patients with HBV infectious liver disease and combined them with data from public databases to analyze the clinical significance of differential metabolites (DMs) and these DM-related enzymes in HCC. This study attempted to elucidate the metabolic mechanism of pathological changes from CHB to HCC and to provide valuable information to find a potential biomarker for the diagnosis and prognosis of HCC.

## RESULTS

### Clinical characteristics of patients

Two hundred six patients were separated into 4 groups, including the healthy (normal control, NC) group (n = 50), CHB group (n = 43), HBV-associated LC group (n = 67) and HBV-associated HCC group (n = 39). All patients were positive for hepatitis B surface antigen (HBsAg), except normal controls. All patients with CHB were treated with antiviral drugs. There were 20 patients with decompensated LC, and all patients with HCC were treated by surgery. The CHB, LC and HCC groups had higher alanine transaminase (ALT), aspartate transaminase (AST), gamma-glutamyl transferase (GGT), and alkaline phosphatase (ALP) levels than the NC group. The serum level of triglyceride (TG) was lower in the LC group than in the CHB group, while the levels of total bile acid (TBA) and total bilirubin (TBIL) were higher in the HCC group than in the LC group. Age, albumin (ALB) and prothrombin time (PT) were not significantly different in patients with various liver diseases (*P* > 0.05). The clinical characteristics of the patients are summarized in Table 1.

**Table 1:** Clinical characteristics of patients.

**Clinical parameters**	**NC (n=50)**	**CHB (n=43)**	**LC (n=67)**	**HCC (n=39)**
Age (year)	38.86±13.56	41.03±12.72	53.53±10.11	56.93±10.55
ALT (U/L)	19.40±6.65	108.31±162.04 **	44.73±38.23 *	48.86±34.32 *
AST (U/L)	21.24±6.56	78.87±126.01 *	63.24±40.20 *	158.00±172.75 **
GGT (U/L)	21.36±9.76	56.62±83.98 *	91.27±131.97 *	155.65±97.77 *
ALP (U/L)	57.71±17.77	89.18±31.20 **	228.53±174.52 **	230.17±117.52 **
ALB (g/L)	43.66±5.21	44.55±4.89	35.40±8.63	33.31±18.44
TG (mmol/L)	0.84±0.31	1.21±0.55 **	1.10±0.73 *	0.83±0.36
TBA (μmol/L)	7.82±1.98	13.89±20.46	69.77±52.36 **	89.25±73.39 **
TBIL (μmol/L)	15.00±3.58	21.97±31.60	33.61±32.24 *	58.80±64.78 *
PT (s)	12.66±0.81	13.39±2.50	14.70±3.29	16.35±3.18
AFP (ng/ml)	---	7.91±9.19	12.95±17.99	249.11±281.38

* *P* < 0.05; ** *P* < 0.01, *vs*. NC.

### Overall metabolic profile of CHB, LC and HCC

**Figure 1:**
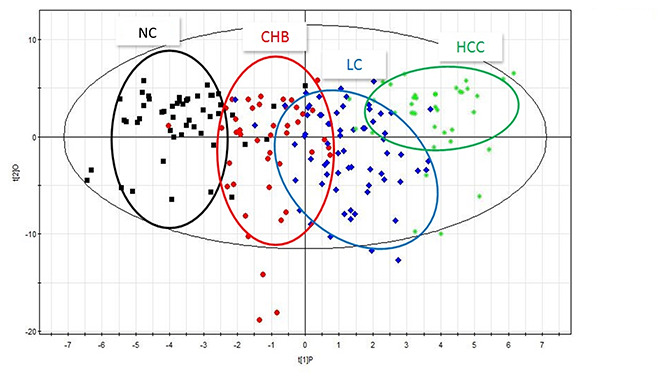
**PLS-DA scatter plots of plasma samples from healthy volunteers, patients with CHB, LC and HCC.** Black represents health, red represents CHB, blue represents LC, green represents HCC.

To investigate the dynamic changes in plasma metabolic profiles from CHB to HCC, the plasma metabolomic data of the NC, CHB, LC and HCC groups were analyzed by partial least square discriminant analysis (PLS-DA). The interpretability of the model on the X axis is 58.1%, that on the Y axis is 83.8%, and the predictive degree is 42.9% (Figure 1). The results showed that the four groups were well differentiated, except for some samples from the CHB group and LC group, and the four groups were arranged from left to right according to the development from healthy to HCC, indicating that the metabolic profiles in patients change gradually with the development of HBV infection-associated diseases.

### Changes of differential metabolites (DMs) in CHB, LC and HCC

**Figure 2:**
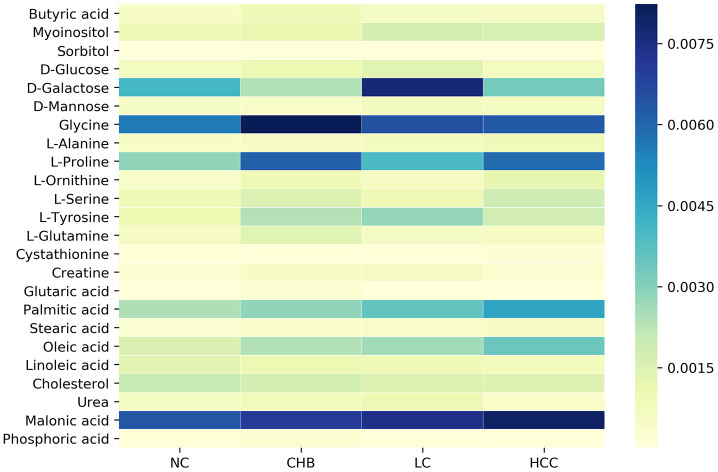
**Dynamic changes of significantly altered metabolites among NC, CHB, LC and HCC.**

To identify the DMs in the NC, CHB, LC and HCC groups, OPLS analysis was performed, and 24 DMs were screened from the NC group and three disease groups by VIP > 1 and *P* < 0.05. We found that the expression levels of various metabolites changed significantly, especially carbohydrates, including glucose, galactose, and mannose, amino acids, including glycine, alanine, proline, and tyrosine, and lipids, including various fatty acids and cholesterol. The dynamic changes of these metabolites in HBV infection disease development are visually shown in Figure 2. These metabolites may be characteristic of the progression of CHB to HCC, including 20, 16, 13, and 16 DMs between NC and CHB, CHB and LC, LC and HCC, HCC and NC, respectively (Supplementary Table 1).

### DM-related metabolic enzyme and pathway enrichment analysis

**Figure 3:**
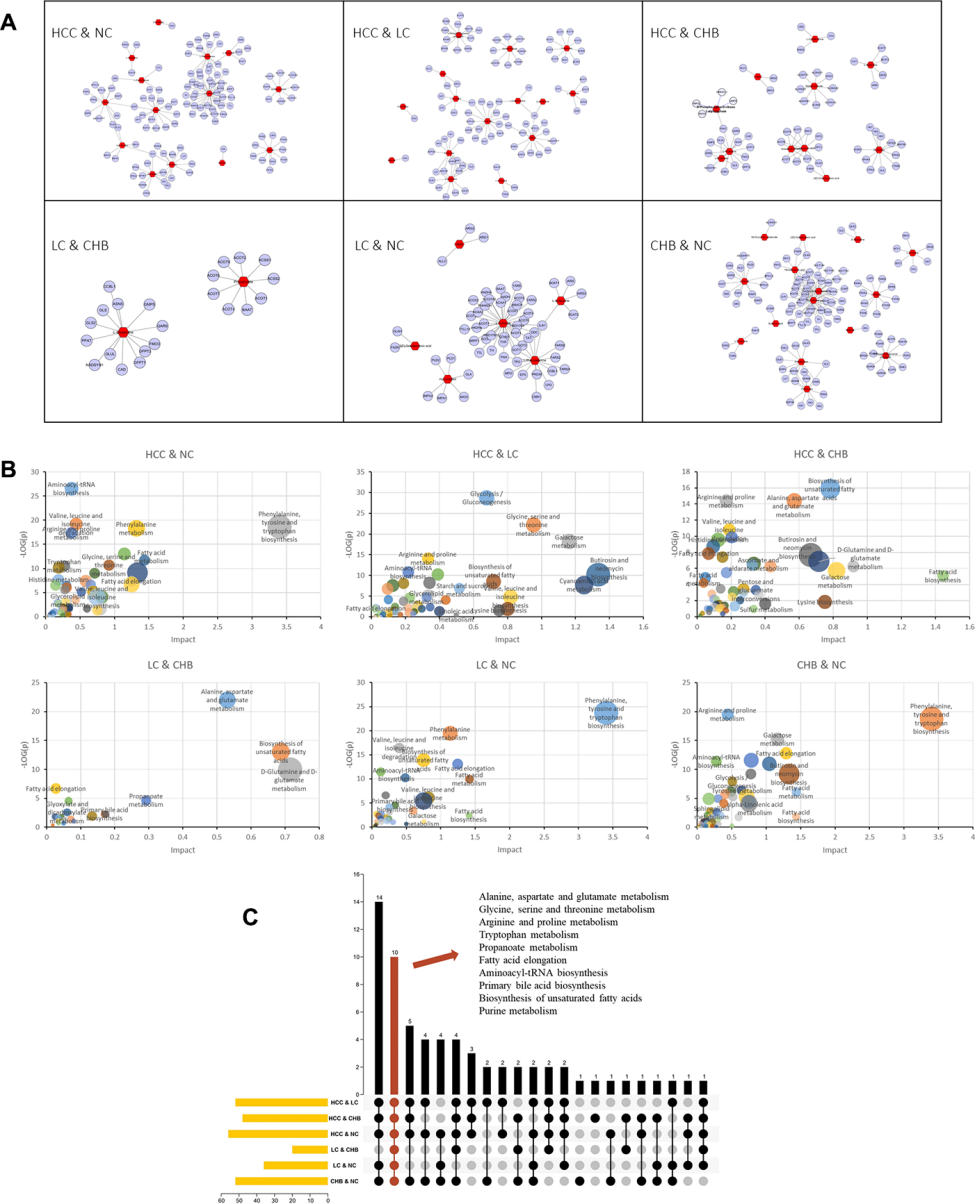
**Genes and KEGG pathways related to DMs.** (**A**) DM-gene interaction networks. The red triangles represent DMs. The blue circles represent metabolic enzyme genes. (**B**) Jointly pathway of DMs and related genes. Abscissa represents the pathway impact. Ordinate represents -log (*p*). (**C**) Upset plot presents the common KEGG pathways among NC, CHB, LC and HCC. The bar chart at the bottom left represents the number of pathways included in each group. The yellow bar chart above represents the number of common pathways in each intersection. Red indicates the intersection of all group.

To identify DM-related metabolic enzymes and pathways, DM-enzyme gene interaction networks (Figure 3A) were built by *MetScape*. According to the networks, 168 metabolic enzyme genes involved in DMs were obtained (Supplementary Table 2). The metabolite-metabolic enzyme gene pairs were input into the *MetaboAnalyst* website to conduct joint pathway analysis (Figure 3B) to simultaneously analyze metabolic enzyme genes and metabolites of interest within the context of metabolic pathways.

To further identify key common pathways from CHB to LC and then to HCC, the intersection of enriched pathways was performed with TBtools. As shown in Figure 3C, alanine, aspartate and glutamate metabolism, glycine, serine and threonine metabolism, arginine and proline metabolism, tryptophan metabolism, propanoate metabolism, fatty acid elongation, aminoacyl-tRNA biosynthesis, primary bile acid biosynthesis, biosynthesis of unsaturated fatty acids and purine metabolism were the common pathways of each group.

### Key metabolic pathways and the mRNA expression of metabolic enzyme genes in HCC

**Figure 4:**
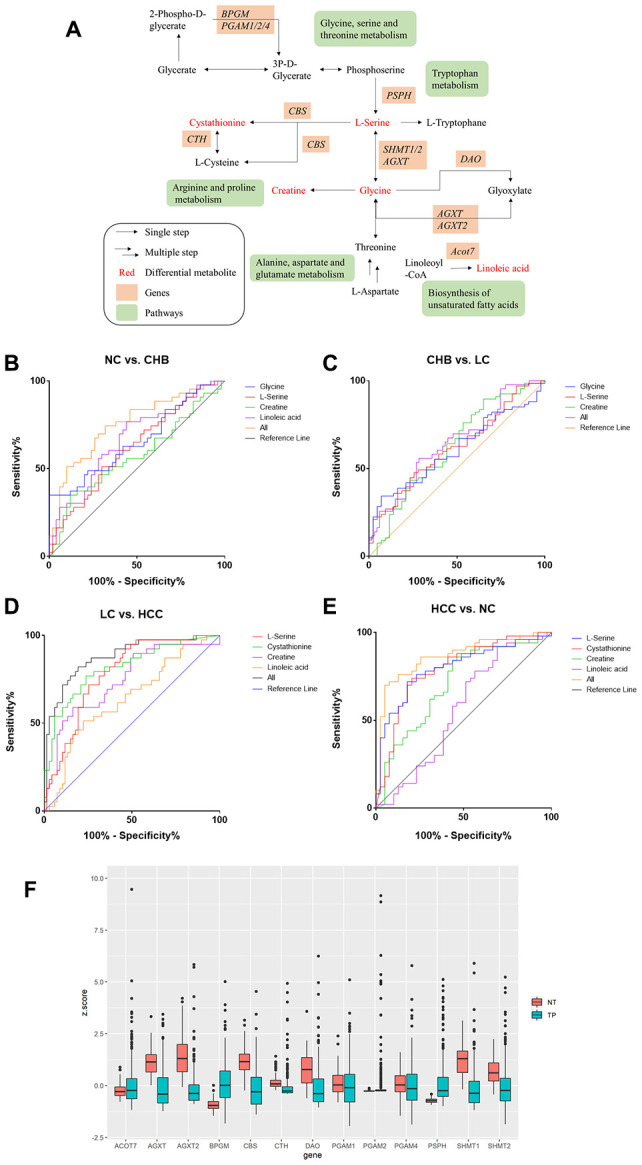
**Metabolic pathway network analysis and expression of candidate metabolic enzyme mRNAs in HCC A.** (**A**) schematic representation of metabolic pathway network. (**B**–**E**). ROC curves of DMs panels between NC and CHB, CHB and LC, LC and HCC, HCC and NC respectively. (**F**) The expression of candidate genes in liver cancer from TCGA-LIHC database (377 patient samples). NT: solid tissue normal, TP: primary solid tumor.

According to the results of the joint pathway analysis above, the intersection of the KEGG pathway is mainly concentrated in amino acid metabolism and lipid metabolism. Furthermore, we combined the DMs and related genes to analyze the metabolic pathway network (Figure 4A) in the development of HCC. The metabolic pathways, including alanine, aspartate and glutamate metabolism, glycine, serine and threonine metabolism, arginine and proline metabolism, tryptophan metabolism and biosynthesis of unsaturated fatty acids, in which L-serine, cystathionine, creatine, glycine and linoleic acid were involved in the development of HCC. Receiver operating characteristic (ROC) curves showed that the panel of L-serine, creatine, glycine and linoleic acid distinguished CHB from NC (AUC=0.753, 95% CI: 0.6524 to 0.8527, *P*<0.0001), L-serine, creatine and glycine distinguished LC from CHB (AUC=0.6439, 95% CI: 0.5380 to 0.7497, *P*=0.0111), L-serine, cystathionine, creatine and linoleic acid distinguished HCC from LC (AUC=0.8772, 95% CI: 0.8073 to 0.9470, *P*<0.0001) and NC (AUC=0.8651, 95% CI: 0.7871 to 0.9431, *P*<0.0001) (Figure 4B–4E), AFP distinguished HCC from LC (AUC= 0.5603, 95% CI: 0.4497 to 0.6708, =0.2547) (Supplementary Figure 1), indicating that the panels of multiple DMs may have greater diagnostic value for distinguishing NC from CHB, CHB from LC, and LC from HCC.

The candidate genes were D-amino-acid oxidase (*DAO*), bisphosphoglycerate mutase (*BPGM*), cystathionine-β-synthase (*CBS*), alanine-glyoxylate aminotransferase (*AGXT*), alanine-glyoxylate aminotransferase-2 (*AGXT2*), cystathionine gamma-lyase (*CTH*), phosphoglycerate mutase 1 (*PGAM1*), phosphoglycerate mutase 2 (*PGAM2*), phosphoglycerate mutase 4 (*PGAM4*), phosphoserine phosphatase (*PSPH*), serine hydroxymethyltransferase 1 (*SHMT1*), serine hydroxymethyltransferase 2 (*SHMT2*) and acyl-CoA thioesterase 7 (*ACOT7*). The expression of these 13 enzyme genes was analyzed using FirebrowseR for a TCGA liver hepatocellular carcinoma (LIHC) dataset (377 patient samples in total). *BPGM*, *PGAM2*, *PSPH*, and *ACOT7* were upregulated in HCC, while *AGXT*, *AGXT2*, *CBS*, *CTH*, *DAO*, *PGAM1*, *PGAM4*, *SHMT1*, and *SHMT2* were downregulated in HCC (Figure 4F).

### Correlations between the expression of candidate metabolic enzyme genes and overall survival (OS) in HCC patients

**Figure 5:**
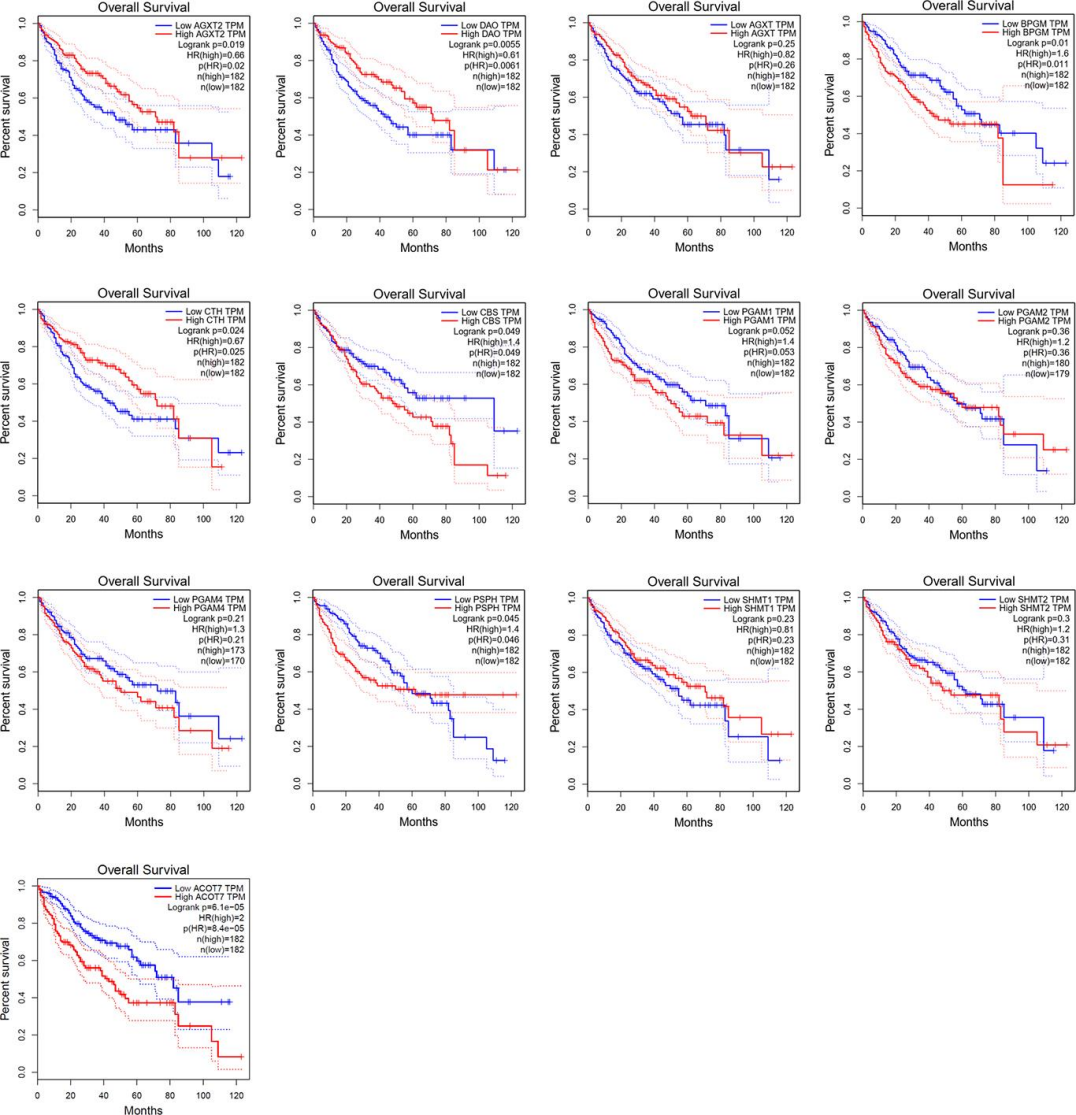
**Survival analysis of candidate genes expressions in HCC patients.**

Kaplan–Meier survival analysis of data from GEPIA revealed that HCC patients with high expression (*n* = 182) of *AGXT2* (HR = 0.66, *P* = 0.019), *DAO* (HR = 0.61, *P* = 0.005), and *CTH* (HR = 0.67, *P* = 0.024) had a better prognosis than patients with low expression (*n* = 182) of *AGXT2*, *DAO*, and *CTH*, while HCC patients with low *BPGM* (HR = 1.6, *P* = 0.010), *CBS* (HR = 1.4, *P* = 0.049), *PSPH* (HR = 1.4, *P* = 0.045), and *ACOT7* (HR = 2, *P* < 0.001) were associated with a longer OS rate (Figure 5). However, *AGXT*, *PGAM1*, *PGAM2*, *PGAM4*, *SHMT1*, and *SHMT2* mRNA expression did not significantly correlate with the OS of HCC patients.

### Correlation between the levels of candidate gene expression and the clinicopathological characteristics in HCC patients

**Figure 6:**
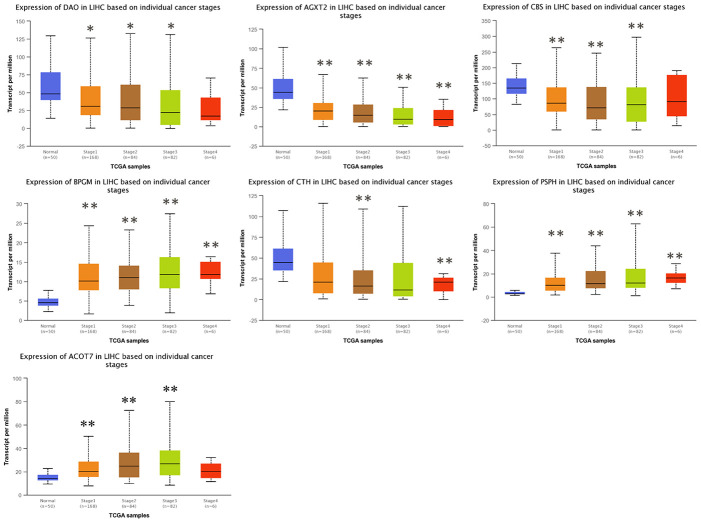
**Correlation between the expressions of *AGXT2*, *DAO*, *CTH*, *BPGM*, *CBS*, *PSPH*, and *ACOT7* mRNAs and Individual cancer stages in HCC.** *P < 0.05, ** *P* < 0.01.

**Figure 7:**
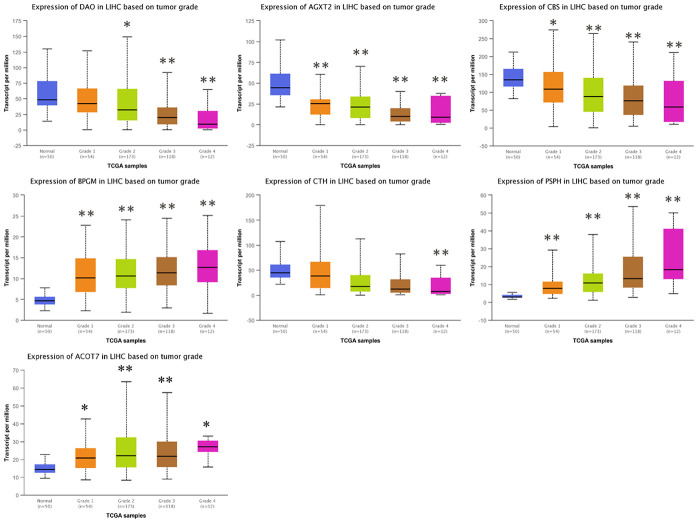
**Correlation between the expressions of *AGXT2*, *DAO*, *CTH*, *BPGM*, *CBS*, *PSPH*, and *ACOT7* mRNAs and Tumor grade in HCC.** *P < 0.05, ** *P* < 0.01.

**Figure 8:**
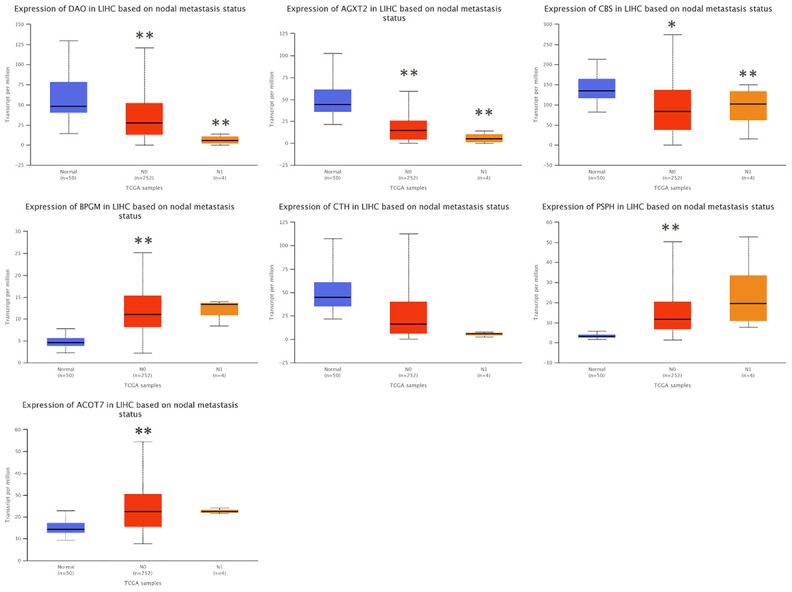
**Correlation between the expressions of *AGXT2*, *DAO*, *CTH*, *BPGM*, *CBS*, *PSPH*, and *ACOT7* mRNAs and Nodal metastasis status in HCC**. *P < 0.05, ** *P* < 0.01.

To investigate the clinical significance of candidate genes, a correlation analysis of clinical parameters with the mRNA expression of *AGXT2*, *DAO*, *CTH*, *BPGM*, *CBS*, *PSPH*, and *ACOT7* in HCC patients was performed with UALCAN. Figures 6–8 shows that mRNA levels of *BPGM* (*P* < < 0.01), *PSPH* (*P* < 0.01), *ACOT7* (*P* < 0.01) and *CBS* (*P* < 0.01) correlated positively with individual cancer stages, tumor grade, and nodal metastasis status. In contrast, *DAO* (*P* < 0.05) and *AGXT2* (*P* < 0.01) mRNA levels were negatively correlated with individual cancer stages, tumor grade, and nodal metastasis status. In addition, there was also a negative correlation between individual cancer stages and tumor grade and the expression of *CTH* (*P* < 0.01), while there was no differential expression of *CTH* in different nodal metastasis statuses.

### Protein expression of *AGXT2*, *DAO*, *CTH*, *BPGM*, *CBS*, *PSPH*, and *ACOT7* in HCC

**Figure 9:**
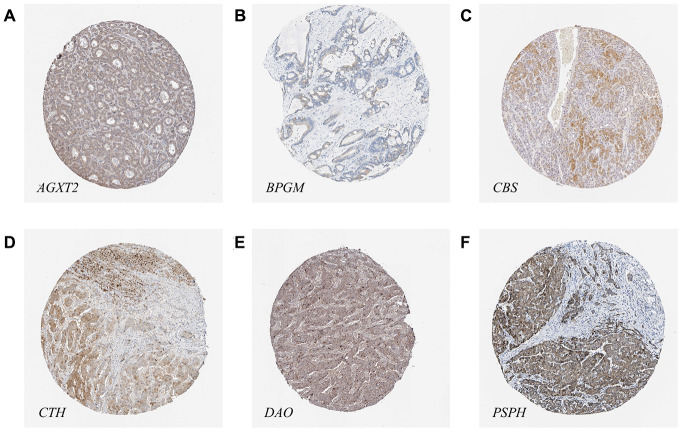
**The protein levels of candidate metabolic enzyme genes in LIHC tissues.** (**A**) *AGXT2* (Antibody HPA037382). (**B**) *BPGM* (Antibody HPA016493). (**C**) *CBS* (Antibody HPA001223). (**D**) *CTH* (Antibody HPA001223). (**E**) *DAO* (Antibody HPA038653). (**F**) *PSPH* (Antibody HPA020376).

The results of immunohistochemical staining from the Human Protein Atlas (HPA) database indicated that there was higher expression of *PSPH* (antibody HPA020376) in LIHC tissues, while there were lower expression levels of *AGXT2* (antibody HPA037382), *BPGM* (antibody HPA016493), *CBS* (antibody HPA001223), *CTH* (antibody HPA001223), and *DAO* (antibody HPA038653) in LIHC tissues (Figure 9). Unfortunately, the results of *Acot7* immunohistochemical staining in LIHC tissues are not available in the HPA database. Except for *BPGM*, the other results were consistent with the mRNA expression levels in HCC.

## DISCUSSION

Liver cancer, as one of the most common neoplasms worldwide, poses a serious threat to human health. Cirrhosis caused by CHB and eventually developing into HCC remains one of the leading causes of HCC and liver failure [15]. A thorough investigation of the metabolism and gene regulation related to the pathological changes in the development of CHB to HCC is of strong significance for further research exploring new strategies for HCC therapy. Many complications of chronic liver disease are closely related to disorders of amino acid metabolism. Research has shown that the disturbance in amino acid metabolism is closely related to the occurrence of various complications of chronic liver disease [16]. Amino acid metabolism and protein metabolism in patients with chronic liver disease are mainly manifested by increased blood ammonia, decreased in plasma albumin and the changes in the amino acid spectrum in the blood [17]. Our study found that the amino acid metabolites, including L-serine, cystathionine, creatine and glycine, detected in HCC patients were upregulated compared with those in healthy volunteers. When liver function is impaired, many enzyme systems are damaged or function is blocked, and a large number of amino acids are released from the liver, resulting in an increase in amino acid concentration in the blood [18].

Free fatty acids are mainly produced by lipolysis of subcutaneous and visceral fat, and some are produced by lipolysis of blood lipase. The liver ingests free fatty acids and oxidizes them for energy supply or for the synthesis of other lipids [19]. In the present study, the blood concentration of linoleic acid, a polyunsaturated fatty acid, in patients with HCC was decreased. In addition, from CHB to LC and to HCC, with the aggravation of liver damage, the content of linoleic acid decreased in the blood of patients. These findings are consistent with earlier reports indicating that linoleic acid can increase apoptosis of rat hepatoma H4IIE cells [20], and unsaturated fatty acids can protect against saturated fatty acid-mediated apoptosis in liver cells [21]. However, other evidence indicated that a polyunsaturated fatty acid can enhance the effects of saturated fatty acids on apoptosis in liver cells [22]. The exact effect and mechanism of linoleic acid in the development of CHB in HCC has not been elucidated to date.

At present, achieving early diagnosis of HCC remains a difficult problem. The sensitivity and specificity of AFP, a tumor marker widely used in the clinic, are not notably high [23], and most patients are already in an advanced stage. Researchers have been looking for new blood markers of HCC, such as AFP-L3, DCP and SCCA [24], and have evaluated serum metabolism during the progression from CHB to HCC [10, 11], but they have not been widely used. In this study, we found that the panel of L-serine, creatine and glycine distinguished LC from CHB, and L-serine, cystathionine, creatine and linoleic acid distinguished HCC from LC and NC, indicating that the panels of multiple DMs may be beneficial to distinguish CHB, LC, and HCC. The diagnostic value of this panel warrants further research.

Monitoring the metabolic enzymes related to DMs links the change of metabolic phenotype and metabolic pathways and provides a direct method to measure the changes of metabolic characteristics on the development of HCC. This study analyzed the joint pathways of DMs and related metabolic enzymes and found that the metabolic pathways intersected by CHB, LC and HCC were primarily concentrated in amino acid metabolism and lipid metabolism. Moreover, the expression levels of *AGXT2*, *DAO*, *CTH*, *BPGM*, *CBS*, *PSPH*, and *ACOT7* mRNAs, as well as the expression of their proteins involved in these metabolic pathways, are closely related to OS and clinicopathological features of HCC patients.

Previous studies have reported that *AGXT2* and *DAO* catalyze the conversion of glyoxylate to glycine [25] and were determined to be related to the risk of metastasis and prognosis of HCC through coexpression network analysis [26]. *BPGM* regulates serine biosynthetic flux by controlling glycolytic intermediate levels [27]. *CTH* breaks down cystathionine into cysteine [28], and deficiencies in *CTH* activity have also been shown to contribute to glutathione depletion in patients with cancer [29]. *CBS* catalyzes L-serine to cystathionine. A study has suggested that positive *CBS* is associated with the clinical severity and poor prognosis in gallbladder cancer [30]. It has been reported that the overexpression of *CBS* leads to drug resistance in HCC cells [31]. *CTH* and *CBS* are also members of the transsulfuration pathway that metabolizes methionine [32], and homozygous (*CTH*−/−) knockout mice displayed acute hepatitis in cases of excessive methionine intake [33]. *PSPH* is responsible for the last step in L-serine formation and is upregulated in Huh7 cells [34]. *ACOT7* catalyzes the hydrolysis of fatty acyl-CoAs to free fatty acids, and low levels of acot7 mRNA extend the overall survival of breast and lung cancer patients [35]. However, the clinical significance of these metabolic enzymes in HCC development has not been fully elucidated. In the present study, metabolic enzymes, such as *AGXT2*, *DAO*, *CTH*, *BPGM*, *CBS*, *PSPH* and *ACOT7*, were found to be associated with the diagnosis and prognosis of HCC. These findings may provide clues for further research to identify biomarkers of diagnosis or prognosis and the pathogenesis of HCC. The changes of these enzymes and metabolites and their metabolisms in liver diseases would be investigated in future studies.

In conclusion, our study suggests that the expressions of plasma L-serine, cystathionine, creatine and glycine were upregulated while linoleic acid was downregulated from CHB, LC to HCC, and the panels of multiple DMs may be helpful to distinguish NC from CHB, CHB from LC, and LC from HCC, respectively. Moreover, the lower expression of *AGXT2*, *DAO* and *CTH* and the higher expression of *BPGM*, *CBS*, *PSPH* and *ACOT7* were associated with poor prognosis for HCC.

## MATERIALS AND METHODS

### Study population

A total of 206 participants were recruited, including 50 normal controls, 43 CHB patients, 67 LC and 39 HCC patients from Shuguang Hospital affiliated with Shanghai University of Traditional Chinese Medicine (Shanghai, China). Specific inclusion and diagnostic criteria refer to our previous work [36]. Patients were excluded if they were younger than 15 or older than 75 years old, were pregnant or breastfeeding, or suffered from other infectious or inflammatory diseases. All methods were carried out in accordance with the approved guidelines (Approval number: 20122062202). The study was approved by the Ethics Committee of Shuguang Hospital and conformed to the ethical guidelines of the Declaration of Helsinki (1964). Written informed consent was signed by all the participants.

### Sample preparation

Venous blood samples were collected before breakfast and then placed at room temperature for 30 min and centrifuged at 12 000 r/min for 10 min at room temperature. The supernatant plasma was separately packed in EP tubes, labeled and stored at -80 °C. Serum biochemical assays including ALT, AST, GGT, ALP, ALB, TG, TBA, TBIL, PT and AFP were performed at Shuguang Hospital using an automatic biochemistry analyzer.

### Metabolomic profiling

An untargeted metabolomics assay was performed in fasting sera from 206 participants using an Agilent 6890 gas chromatography system coupled with a 5975B mass spectrometer (Agilent Technologies, USA). The pretreatment method of the plasma sample and chromatographic separation were described in our previous work [37]. The mass spectrometry scan range is 30–550 m/z. Ion source temperature and quadrupole temperature, 230 °C and 150 °C, respectively. Identification and relative quantification of metabolites were carried out using the Agilent Mass Hunter Workstation (Agilent Technologies, USA).

### Bioinformatic analysis

Differential metabolite-gene networks were constructed by *MetScape* [38] running on Cystoscape (version 3.6.1) [39]. Joint pathway analysis and visualization of metabolic pathways were generated by using *MetaboAnalyst* (http://www.metaboanalyst.ca) [40]. UpSet plot, a novel technique to visualize interactive sets, was used to analyze the intersections of pathways among NC, CHB, LC and HCC by using TBtools [41]. Data from TCGA for the LIHC cohort were accessed via FirebrowseR [42], an R client to the Broad Institute’s RESTful Firehose Pipeline. Gene expression data were visualized in R using the ggplot2 package. Kaplan–Meier survival analysis was performed with GEPIA (http://gepia.cancer-pku.cn) [43] to evaluate the prognostic value of distinct enzyme gene expression in HCC patients. The relationships between mRNA levels of enzyme genes and clinicopathological features were analyzed on the UALCAN website (http://ualcan.path.uab.edu) [44]. The immunohistochemical staining results of candidate genes in LIHC were retrieved from the HPA database (https://www.proteinatlas.org/).

### Data analysis

SPSS 25.0 (Chicago, IL, USA) was used to carry out statistical analysis, and GraphPad Prism (version 6.0.7, San Diego, California, USA) was used for statistical charts and ROC curves. Normalized data were imported into SIMCA-P (version 11.0, Umetric, Umea, Sweden) for PLS-DA, OPLS and the calculation of variable importance in the projection (VIP). The Kruskal-Wallis test is used for nonparametric comparison of continuous variables. Fisher’s exact test for categorical variables was used for multiple comparison correction and adjusted by the FDR method.

## Supplementary Material

Supplementary Figure 1

Supplementary Table 1

Supplementary Table 2
