# Supplementary figures and images for: Analysis of plasma metabolic profile, characteristics and enzymes in the progression from chronic hepatitis B to hepatocellular carcinoma
Source: Aging (Albany NY). 2020 Jul 23;12(14):14949–65. doi: 10.18632/aging.103554 (PMC7425494; doi:10.18632/aging.103554)

SUPPLEMENTARY FIGURE

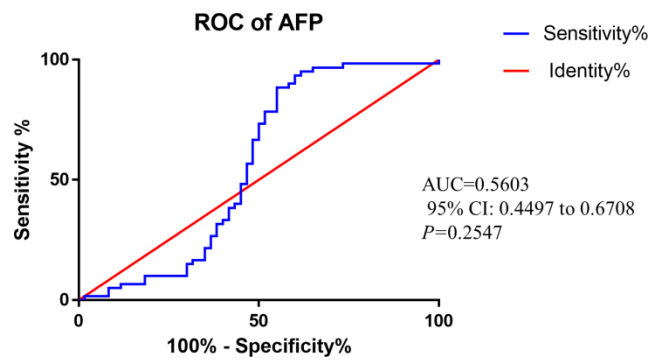

Supplementary Figure 1. ROC curve of AFP between LC and HCC.

Supplement: Supplementary Figure 1 [file aging-12-103554-s003..pdf]
