# Supplementary material for: Analysis of plasma metabolic profile, characteristics and enzymes in the progression from chronic hepatitis B to hepatocellular carcinoma
Source: Aging (Albany NY). 2020 Jul 23;12(14):14949–65. doi: 10.18632/aging.103554 (PMC7425494; doi:10.18632/aging.103554)
Supplement: Supplementary Table 1 [file aging-12-103554-s002..docx]

| NC | CHB |  | CHB | LC |  | LC | HCC |  | HCC | NC |
| --- | --- | --- | --- | --- | --- | --- | --- | --- | --- | --- |
| Butyric acid | |  | Sorbitol | |  | D-Glucose | |  | Myoinositol | |
| 355095.6 | 247458.8 |  | 59.16544 | 6831.144 |  | 621321.5 | 537207.1 |  | 207350 | 265702.3 |
| 86268.42 | 306267.8 |  | 12366.9 | 16528.78 |  | 62046.78 | 97340.14 |  | 234639.8 | 140306.7 |
| 114183.7 | 151396.2 |  | 10344.85 | 15339.78 |  | 272474.9 | 35384.88 |  | 189480.5 | 161330.3 |
| 78439.77 | 132386.7 |  | 11711.69 | 23749.22 |  | 306060.9 | 483926.9 |  | 667930.5 | 172516.5 |
| 78581.32 | 236171.9 |  | 15324.25 | 20895.3 |  | 173370.7 | 674348.7 |  | 958469.3 | 204472.8 |
| 81981.47 | 171144.3 |  | 32144.02 | 12044.03 |  | 209264.3 | 150230.8 |  | 1765324 | 98948.93 |
| 111967.2 | 8935.614 |  | 5861.224 | 14054.32 |  | 46517.01 | 642.7496 |  | 254262.9 | 158771.5 |
| 102679.5 | 11886.45 |  | 8011.119 | 13493.07 |  | 215583.7 | 128205.2 |  | 247983.9 | 164825.4 |
| 94785 | 21670.95 |  | 6650.87 | 6407.514 |  | 39944.07 | 291075.7 |  | 597939.8 | 105969.2 |
| 21775.9 | 20058.36 |  | 17431.12 | 0 |  | 449506.7 | 264486.9 |  | 218284.1 | 166928 |
| 9805.473 | 38342.66 |  | 16532.66 | 21518.59 |  | 274889.4 | 428719.6 |  | 195098.7 | 92055.77 |
| 286841.9 | 116270.1 |  | 9409.485 | 58.92184 |  | 331432 | 365005.8 |  | 220453.1 | 244005.1 |
| 24347.27 | 47049.75 |  | 12672.14 | 10783.27 |  | 110771.2 | 187201.3 |  | 316632.1 | 200274.3 |
| 56125.32 | 69490.78 |  | 777.86 | 897.6892 |  | 225825.8 | 172476.3 |  | 253567.4 | 124851.8 |
| 47418.83 | 131888.8 |  | 127.3296 | 11701.21 |  | 173988.3 | 62039.25 |  | 281873.1 | 168046.9 |
| 230670.8 | 102245.3 |  | 12606.24 | 15884.17 |  | 191156.3 | 44446.76 |  | 272818.5 | 187852.8 |
| 108831.2 | 89086.03 |  | 17853.35 | 1018.005 |  | 40990.11 | 104264.8 |  | 234247.4 | 226141.7 |
| 148377.6 | 134778.4 |  | 15137.06 | 13425.16 |  | 258313.7 | 59965.93 |  | 276866.3 | 192080.6 |
| 297414.6 | 143441.1 |  | 11754.07 | 11490.33 |  | 366455.2 | 45349.91 |  | 268487.6 | 113660.9 |
| 70866.08 | 167840.8 |  | 18830.95 | 17587.12 |  | 265016.4 | 209824.6 |  | 182631.4 | 248867.2 |
| 152978.9 | 156761.2 |  | 6867.526 | 12496.46 |  | 323764.2 | 52229.35 |  | 232028.7 | 384390.5 |
| 74208.42 | 510940.4 |  | 20874.25 | 670.2305 |  | 455975 | 46988.04 |  | 324295.3 | 197924.8 |
| 168216.9 | 73712.55 |  | 4975.369 | 3375.254 |  | 11036.93 | 206481.4 |  | 204559.4 | 151270.1 |
| 169820.6 | 165485.8 |  | 11802.06 | 15587.78 |  | 727393.9 | 28504.73 |  | 315887.4 | 127260.3 |
| 154268.7 | 175889 |  | 11102.5 | 11024.76 |  | 207293.3 | 172634.9 |  | 344713.5 | 217530.1 |
| 183424.6 | 233770.1 |  | 10638.21 | 19814.45 |  | 191042.5 | 143547.8 |  | 215213.9 | 119210.7 |
| 191761.3 | 108210.8 |  | 7029.348 | 4669.744 |  | 1167582 | 158491.9 |  | 351048.9 | 146544.3 |
| 108598.7 | 325696.4 |  | 6703.049 | 56441.52 |  | 378098.7 | 1929.723 |  | 1230568 | 160836.5 |
| 194617.2 | 273706.2 |  | 11701.72 | 13252.53 |  | 435181.7 | 63559.14 |  | 250816.2 | 215391.2 |
| 210560.7 | 237447.7 |  | 17230.48 | 13372.06 |  | 168233.1 | 7717.043 |  | 439112.8 | 150485.7 |
| 180430.8 | 237240.3 |  | 0 | 22874.61 |  | 74631.63 | 139021.4 |  | 341912.6 | 166353 |
| 29213.01 | 142660.7 |  | 570.9966 | 24077.55 |  | 141718.2 | 18829.6 |  | 480132.4 | 209306 |
| 163583.7 | 618.0184 |  | 0 | 10867.19 |  | 231594 | 435501.3 |  | 389238.9 | 159541.8 |
| 199428.4 | 119981.8 |  | 550.3851 | 12435.89 |  | 214141.9 | 153709.7 |  | 287986.1 | 180478.5 |
| 122389.3 | 182484.2 |  | 13899.24 | 15277.97 |  | 206253.3 | 288984.1 |  | 291769.3 | 165366.7 |
| 342872.2 | 177835.1 |  | 1252.011 | 30375.49 |  | 519312.6 | 103741.5 |  | 236588 | 288516.1 |
| 136.5612 | 929764.1 |  | 107.1316 | 17932.16 |  | 447092.3 | 199198.1 |  | 289238.3 | 177415.4 |
| 60.54567 | 679115.6 |  | 3758.164 | 15301.26 |  | 362224.7 | 110391.7 |  | 333258.7 | 161594.1 |
| 282.5468 | 22887.98 |  | 2388.206 | 12961.38 |  | 361938.6 | 30179.82 |  | 211229.5 | 237576.8 |
| 287.4677 | 75511.62 |  | 58.56329 | 21320.11 |  | 369524.6 |  |  |  | 254507.5 |
| 123809.6 | 60671.67 |  | 20209.45 | 9679.587 |  | 223121.2 |  |  |  | 158855.3 |
| 600.67 | 214803.1 |  | 33011.79 | 5211.753 |  | 20073.36 |  |  |  | 182757.8 |
| 127.6909 | 127923.2 |  | 14179.29 | 15934.09 |  | 221070.7 |  |  |  | 179934.7 |
| 73907.37 |  |  |  | 7140.125 |  | 631940.5 |  |  |  | 176139.8 |
| 161972.4 |  |  |  | 22951.25 |  | 644086.6 |  |  |  | 206917.1 |
| 24014.29 |  |  |  | 23285.07 |  | 643489.2 |  |  |  | 163621.5 |
| 53848.1 |  |  |  | 7996.767 |  | 245609.9 |  |  |  | 185498.9 |
| 44784.54 |  |  |  | 8642.473 |  | 236214.6 |  |  |  | 127332.2 |
| 81432.31 |  |  |  | 13721.45 |  | 228272.3 |  |  |  | 256920.1 |
| 46226.59 |  |  |  | 15290.87 |  | 252992.2 |  |  |  | 144455.3 |
|  |  |  |  | 43993.99 |  | 110114.6 |  |  |  |  |
| Myoinositol | |  |  | 24258.01 |  | 59018.37 |  |  | Sorbitol | |
| 265702.3 | 231124 |  |  | 12273.33 |  | 8276.984 |  |  | 1173.094 | 0 |
| 140306.7 | 168388.8 |  |  | 19444.52 |  | 66639.87 |  |  | 18533.28 | 9531.283 |
| 161330.3 | 208111.8 |  |  | 12184.2 |  | 864848.3 |  |  | 16281.9 | 13014.7 |
| 172516.5 | 150636.4 |  |  | 36786.83 |  | 532674.4 |  |  | 37761.25 | 13263.64 |
| 204472.8 | 208944.9 |  |  | 35654.69 |  | 371990.8 |  |  | 24600.25 | 21605.92 |
| 98948.93 | 166011.3 |  |  | 216.1779 |  | 610575.3 |  |  | 13302.98 | 9904.862 |
| 158771.5 | 130180.4 |  |  | 8860.965 |  | 234979.3 |  |  | 0 | 12838.44 |
| 164825.4 | 111553.2 |  |  | 686.6828 |  | 496587.6 |  |  | 12488.17 | 13784.15 |
| 105969.2 | 170504 |  |  | 3683.845 |  | 194456.4 |  |  | 21868.92 | 12782.05 |
| 166928 | 218581.8 |  |  | 3629.101 |  | 424286.7 |  |  | 2440.518 | 10032.6 |
| 92055.77 | 329650.2 |  |  | 1373.128 |  | 258323.7 |  |  | 0 | 9252.692 |
| 244005.1 | 195759.3 |  |  | 614.9186 |  | 172766.1 |  |  | 28870.25 | 11985.69 |
| 200274.3 | 217949.6 |  |  | 31861.58 |  | 948108.8 |  |  | 20506.87 | 11271.99 |
| 124851.8 | 211360.8 |  |  | 16385.2 |  | 306717.6 |  |  | 15836.81 | 0 |
| 168046.9 | 316786.6 |  |  | 16085.87 |  | 120100.3 |  |  | 20110.95 | 13771.19 |
| 187852.8 | 236999.1 |  |  |  |  |  |  |  | 14041.13 | 14920.89 |
| 226141.7 | 314126.2 |  | D-Glucose | |  | D-Galactose | |  | 15776.25 | 15122.15 |
| 192080.6 | 232234.4 |  | 661443.4 | 621321.5 |  | 4587729 | 433736.6 |  | 14662.85 | 15179.76 |
| 113660.9 | 161294.5 |  | 167493.1 | 62046.78 |  | 681795.4 | 703961.6 |  | 22707.08 | 704.6597 |
| 248867.2 | 182778 |  | 227240.3 | 272474.9 |  | 504648 | 737300.6 |  | 13722.49 | 6758.063 |
| 384390.5 | 182981.8 |  | 53502.3 | 306060.9 |  | 639467.2 | 583355.5 |  | 13891.71 | 10348.3 |
| 197924.8 | 202651.8 |  | 242728.5 | 173370.7 |  | 666549.1 | 3863975 |  | 27293.52 | 8458.087 |
| 151270.1 | 123019.8 |  | 137193 | 209264.3 |  | 458950.5 | 484210.6 |  | 15271.28 | 10380.42 |
| 127260.3 | 232768 |  | 239682.1 | 46517.01 |  | 559585.8 | 560.0191 |  | 17936.79 | 13456.66 |
| 217530.1 | 234356.1 |  | 84967.62 | 215583.7 |  | 521904.5 | 510200.1 |  | 19177.51 | 15591.94 |
| 119210.7 | 344841.3 |  | 146373.9 | 39944.07 |  | 463823 | 643652.6 |  | 14757.97 | 16974.41 |
| 146544.3 | 201554.6 |  | 251512.1 | 449506.7 |  | 2836765 | 427292.2 |  | 35757.14 | 3590.299 |
| 160836.5 | 243995.9 |  | 242799.2 | 274889.4 |  | 1423317 | 2650592 |  | 12125.12 | 10738.86 |
| 215391.2 | 215695.9 |  | 38214.27 | 331432 |  | 425639.5 | 558839.1 |  | 14783.16 | 14012.74 |
| 150485.7 | 270664.4 |  | 196226 | 110771.2 |  | 502986.6 | 575390.7 |  | 16927.75 | 13035.89 |
| 166353 | 240290.4 |  | 49994.88 | 225825.8 |  | 482202 | 517491.7 |  | 22924.76 | 11924.48 |
| 209306 | 283924.4 |  | 2763.208 | 173988.3 |  | 476733.7 | 705979 |  | 11528.24 | 13939.84 |
| 159541.8 | 326118.5 |  | 232222 | 191156.3 |  | 527743.3 | 558253 |  | 12205.75 | 11530.18 |
| 180478.5 | 210087.1 |  | 298811.5 | 40990.11 |  | 332602.6 | 623732.4 |  | 38239.49 | 11111.38 |
| 165366.7 | 258552 |  | 212659.8 | 258313.7 |  | 516707.3 | 551557.6 |  | 25658.63 | 9512.884 |
| 288516.1 | 308506.9 |  | 59652.47 | 366455.2 |  | 474514.1 | 743721.5 |  | 42419.5 | 16867.6 |
| 177415.4 | 284617.8 |  | 245449.3 | 265016.4 |  | 538017.4 | 558909.8 |  | 15132.44 | 2966.597 |
| 161594.1 | 240898.3 |  | 98796.39 | 323764.2 |  | 433776.1 | 640316.5 |  | 43251.8 | 4097.345 |
| 237576.8 | 267419.5 |  | 527904.9 | 455975 |  | 398876.8 | 854589.3 |  | 21271.26 | 1399.989 |
| 254507.5 | 184600.9 |  | 51356.34 | 11036.93 |  | 202229.1 | 531484 |  |  | 6869.758 |
| 158855.3 | 239921.7 |  | 17586.62 | 727393.9 |  | 4638794 | 694175.5 |  |  | 12605.97 |
| 182757.8 | 278073 |  | 232576.3 | 207293.3 |  | 587809.8 | 773391.2 |  |  | 5828.098 |
| 179934.7 | 147020.4 |  | 50788.13 | 191042.5 |  | 761443.4 | 726343.3 |  |  | 2054.521 |
| 176139.8 |  |  | 12100.78 | 1167582 |  | 7948495 | 1579130 |  |  | 10468.88 |
| 206917.1 |  |  | 71937.05 | 378098.7 |  | 4299.417 | 218733.9 |  |  | 12080.76 |
| 163621.5 |  |  | 46747.03 | 435181.7 |  | 3446148 | 292403.2 |  |  | 17024.77 |
| 185498.9 |  |  | 167159.3 | 168233.1 |  | 902672.5 | 239986.1 |  |  | 11520.83 |
| 127332.2 |  |  | 2333.257 | 74631.63 |  | 283207 | 648881.4 |  |  | 13171.4 |
| 256920.1 |  |  | 6126.809 | 141718.2 |  | 712577.5 | 210363.9 |  |  | 14211.83 |
| 144455.3 |  |  | 1829.345 | 231594 |  | 1525768 | 2977435 |  |  | 11691.54 |
|  |  |  | 1716.814 | 214141.9 |  | 447904.1 | 1112545 |  |  |  |
| D-Glucose | |  | 64462.5 | 206253.3 |  | 1318953 | 598887.4 |  | D-Glucose | |
| 609333.9 | 661443.4 |  | 18937.73 | 519312.6 |  | 4125317 | 1251233 |  | 537207.1 | 609333.9 |
| 101559.2 | 167493.1 |  | 1825.356 | 447092.3 |  | 3532784 | 547981.7 |  | 97340.14 | 101559.2 |
| 336483.4 | 227240.3 |  | 34036.37 | 362224.7 |  | 2529285 | 1367523 |  | 35384.88 | 336483.4 |
| 660439.9 | 53502.3 |  | 113839.3 | 361938.6 |  | 2392121 | 839264.2 |  | 483926.9 | 660439.9 |
| 30913.28 | 242728.5 |  | 350708.7 | 369524.6 |  | 2449754 |  |  | 674348.7 | 30913.28 |
| 82194.02 | 137193 |  | 411618.6 | 223121.2 |  | 1266303 |  |  | 150230.8 | 82194.02 |
| 47677.05 | 239682.1 |  | 512047.6 | 20073.36 |  | 286867.8 |  |  | 642.7496 | 47677.05 |
| 294203.2 | 84967.62 |  | 113476.4 | 221070.7 |  | 1285124 |  |  | 128205.2 | 294203.2 |
| 117433.6 | 146373.9 |  |  | 631940.5 |  | 4425199 |  |  | 291075.7 | 117433.6 |
| 390318.3 | 251512.1 |  |  | 644086.6 |  | 5269955 |  |  | 264486.9 | 390318.3 |
| 80643.69 | 242799.2 |  |  | 643489.2 |  | 4982365 |  |  | 428719.6 | 80643.69 |
| 308579.2 | 38214.27 |  |  | 245609.9 |  | 1352096 |  |  | 365005.8 | 308579.2 |
| 293366.1 | 196226 |  |  | 236214.6 |  | 1377183 |  |  | 187201.3 | 293366.1 |
| 16457.3 | 49994.88 |  |  | 228272.3 |  | 1211673 |  |  | 172476.3 | 16457.3 |
| 302883.6 | 2763.208 |  |  | 252992.2 |  | 89943.95 |  |  | 62039.25 | 302883.6 |
| 437359.8 | 232222 |  |  | 110114.6 |  | 1022557 |  |  | 44446.76 | 437359.8 |
| 331270.5 | 298811.5 |  |  | 59018.37 |  | 285312 |  |  | 104264.8 | 331270.5 |
| 410953 | 212659.8 |  |  | 8276.984 |  | 312580.8 |  |  | 59965.93 | 410953 |
| 55237.24 | 59652.47 |  |  | 66639.87 |  | 201790.3 |  |  | 45349.91 | 55237.24 |
| 188651.8 | 245449.3 |  |  | 864848.3 |  | 6452943 |  |  | 209824.6 | 188651.8 |
| 464213 | 98796.39 |  |  | 532674.4 |  | 5249426 |  |  | 52229.35 | 464213 |
| 215087.3 | 527904.9 |  |  | 371990.8 |  | 2054262 |  |  | 46988.04 | 215087.3 |
| 208582.1 | 51356.34 |  |  | 610575.3 |  | 4998586 |  |  | 206481.4 | 208582.1 |
| 238721.3 | 17586.62 |  |  | 234979.3 |  | 1346774 |  |  | 28504.73 | 238721.3 |
| 210717.3 | 232576.3 |  |  | 496587.6 |  | 3383756 |  |  | 172634.9 | 210717.3 |
| 222532.6 | 50788.13 |  |  | 194456.4 |  | 1104504 |  |  | 143547.8 | 222532.6 |
| 117976.1 | 12100.78 |  |  | 424286.7 |  | 2736905 |  |  | 158491.9 | 117976.1 |
| 53226.77 | 71937.05 |  |  | 258323.7 |  | 1598166 |  |  | 1929.723 | 53226.77 |
| 107293.7 | 46747.03 |  |  | 172766.1 |  | 120565.9 |  |  | 63559.14 | 107293.7 |
| 254234.9 | 167159.3 |  |  | 948108.8 |  | 5824509 |  |  | 7717.043 | 254234.9 |
| 119330.2 | 2333.257 |  |  | 306717.6 |  | 536301.3 |  |  | 139021.4 | 119330.2 |
| 156219 | 6126.809 |  |  | 120100.3 |  | 639104.5 |  |  | 18829.6 | 156219 |
| 37143.89 | 1829.345 |  |  |  |  |  |  |  | 435501.3 | 37143.89 |
| 277901 | 1716.814 |  | D-Galactose | |  | L-Alanine | |  | 153709.7 | 277901 |
| 62671.11 | 64462.5 |  | 4522558 | 4587729 |  | 182821.9 | 118185.4 |  | 288984.1 | 62671.11 |
| 323008.5 | 18937.73 |  | 476249.7 | 681795.4 |  | 137251.8 | 105911.9 |  | 103741.5 | 323008.5 |
| 250985 | 1825.356 |  | 496588.3 | 504648 |  | 110068.8 | 164190 |  | 199198.1 | 250985 |
| 144088.5 | 34036.37 |  | 552857.1 | 639467.2 |  | 65784.13 | 344710.5 |  | 110391.7 | 144088.5 |
| 173973.4 | 113839.3 |  | 595391.6 | 666549.1 |  | 150692.2 | 308256.3 |  | 30179.82 | 173973.4 |
| 133695.8 | 350708.7 |  | 1141898 | 458950.5 |  | 123284.2 | 43381.53 |  |  | 133695.8 |
| 142810.3 | 411618.6 |  | 338205.7 | 559585.8 |  | 105679.1 | 133120.2 |  |  | 142810.3 |
| 293657.5 | 512047.6 |  | 385605.6 | 521904.5 |  | 110394.8 | 195091.3 |  |  | 293657.5 |
| 181469 | 113476.4 |  | 351899.1 | 463823 |  | 85395.52 | 179112.9 |  |  | 181469 |
| 200374 |  |  | 518664.4 | 2836765 |  | 180174.7 | 67370.41 |  |  | 200374 |
| 500650 |  |  | 510758.9 | 1423317 |  | 131057.9 | 123834.5 |  |  | 500650 |
| 324678.4 |  |  | 493943.7 | 425639.5 |  | 131458.8 | 253365.5 |  |  | 324678.4 |
| 277641 |  |  | 462300.4 | 502986.6 |  | 165322.4 | 267921.9 |  |  | 277641 |
| 102086.7 |  |  | 223411.4 | 482202 |  | 173319.8 | 245801.6 |  |  | 102086.7 |
| 478728 |  |  | 1473.39 | 476733.7 |  | 161294.2 | 355882.4 |  |  | 478728 |
| 171034.6 |  |  | 479725.2 | 527743.3 |  | 186369.1 | 259699.2 |  |  | 171034.6 |
|  |  |  | 497797.4 | 332602.6 |  | 76839.53 | 187520.5 |  |  |  |
| D-Galactose | |  | 515611.4 | 516707.3 |  | 208653.2 | 228086.7 |  | L-Alanine | |
| 4169692 | 4522558 |  | 480016.4 | 474514.1 |  | 183929.9 | 96677.33 |  | 118185.4 | 226931.1 |
| 522350.4 | 476249.7 |  | 584682.9 | 538017.4 |  | 217022 | 222393.2 |  | 105911.9 | 91290.62 |
| 502869.1 | 496588.3 |  | 585695 | 433776.1 |  | 198458 | 191609.4 |  | 164190 | 142004.7 |
| 4504797 | 552857.1 |  | 542401.8 | 398876.8 |  | 108346.4 | 256481.9 |  | 344710.5 | 183672.2 |
| 830722 | 595391.6 |  | 482831.9 | 202229.1 |  | 219658.7 | 195656.8 |  | 308256.3 | 175113.2 |
| 556741.6 | 1141898 |  | 651149.6 | 4638794 |  | 120231.2 | 157254.8 |  | 43381.53 | 106236 |
| 607114.4 | 338205.7 |  | 1461290 | 587809.8 |  | 73213.46 | 191597 |  | 133120.2 | 99510 |
| 556989.7 | 385605.6 |  | 217133.8 | 761443.4 |  | 1154.31 | 108657.6 |  | 195091.3 | 121075.4 |
| 551645.1 | 351899.1 |  | 260998.3 | 7948495 |  | 82273.67 | 530455.5 |  | 179112.9 | 103193.8 |
| 365813.2 | 518664.4 |  | 181828.4 | 4299.417 |  | 18491.16 | 904200.9 |  | 67370.41 | 178224.9 |
| 434537.5 | 510758.9 |  | 244669.8 | 3446148 |  | 139013.4 | 147872.6 |  | 123834.5 | 103727.5 |
| 402248.9 | 493943.7 |  | 854829.6 | 902672.5 |  | 79408.45 | 167616.5 |  | 253365.5 | 199097.7 |
| 418068.2 | 462300.4 |  | 1157.617 | 283207 |  | 45286.37 | 273961.1 |  | 267921.9 | 130135.8 |
| 449622.8 | 223411.4 |  | 2508.844 | 712577.5 |  | 226365.3 | 210266.5 |  | 245801.6 | 139982.5 |
| 483416.1 | 1473.39 |  | 1230.855 | 1525768 |  | 86527.58 | 2861.967 |  | 355882.4 | 138618.3 |
| 452510.9 | 479725.2 |  | 110172.7 | 447904.1 |  | 204068.3 | 268989.4 |  | 259699.2 | 232504.2 |
| 489935 | 497797.4 |  | 937356.6 | 1318953 |  | 31527.94 | 226568.2 |  | 187520.5 | 293291.2 |
| 451551.7 | 515611.4 |  | 103086.6 | 4125317 |  | 215164.6 | 212136.7 |  | 228086.7 | 270365.5 |
| 237004.9 | 480016.4 |  | 2572.783 | 3532784 |  | 190945.1 | 164468.7 |  | 96677.33 | 152440.8 |
| 382139.1 | 584682.9 |  | 205629 | 2529285 |  | 155275.4 | 1652.282 |  | 222393.2 | 235920.1 |
| 346518 | 585695 |  | 747126.7 | 2392121 |  | 1928.188 | 110060.3 |  | 191609.4 | 224076.4 |
| 379484.6 | 542401.8 |  | 385973.2 | 2449754 |  | 188847.9 |  |  | 256481.9 | 297467.8 |
| 465014.5 | 482831.9 |  | 478991.5 | 1266303 |  | 111624 |  |  | 195656.8 | 235879.3 |
| 515333.9 | 651149.6 |  | 569336.9 | 286867.8 |  | 60952.31 |  |  | 157254.8 | 20881.17 |
| 569744.7 | 1461290 |  | 548508 | 1285124 |  | 118745 |  |  | 191597 | 1259.674 |
| 585036.9 | 217133.8 |  |  | 4425199 |  | 99360.95 |  |  | 108657.6 | 132065.4 |
| 549235.9 | 260998.3 |  |  | 5269955 |  | 2405.85 |  |  | 530455.5 | 26741.64 |
| 564352.1 | 181828.4 |  |  | 4982365 |  | 2164.448 |  |  | 904200.9 | 17105.62 |
| 625357.5 | 244669.8 |  |  | 1352096 |  | 1314.12 |  |  | 147872.6 | 2279.932 |
| 1636912 | 854829.6 |  |  | 1377183 |  | 1822.906 |  |  | 167616.5 | 99910.61 |
| 643049.6 | 1157.617 |  |  | 1211673 |  | 2446.194 |  |  | 273961.1 | 101511.8 |
| 846826.8 | 2508.844 |  |  | 89943.95 |  | 1782.823 |  |  | 210266.5 | 102664.8 |
| 251600.4 | 1230.855 |  |  | 1022557 |  | 248374.3 |  |  | 2861.967 | 113503.1 |
| 413441.6 | 110172.7 |  |  | 285312 |  | 174096.8 |  |  | 268989.4 | 250717.7 |
| 241331.7 | 937356.6 |  |  | 312580.8 |  | 27295.66 |  |  | 226568.2 | 81800.64 |
| 2235112 | 103086.6 |  |  | 201790.3 |  | 273141.9 |  |  | 212136.7 | 119218.4 |
| 1606603 | 2572.783 |  |  | 6452943 |  | 109403.3 |  |  | 164468.7 | 2391.582 |
| 789777.5 | 205629 |  |  | 5249426 |  | 195066.2 |  |  | 1652.282 | 2047.638 |
| 1072991 | 747126.7 |  |  | 2054262 |  | 57156.01 |  |  | 110060.3 | 2971.244 |
| 722017.9 | 385973.2 |  |  | 4998586 |  | 6448.67 |  |  |  | 2410.25 |
| 883683 | 478991.5 |  |  | 1346774 |  | 2137.993 |  |  |  | 103780.6 |
| 1886507 | 569336.9 |  |  | 3383756 |  | 4492.577 |  |  |  | 2286.206 |
| 1028732 | 548508 |  |  | 1104504 |  | 3577.81 |  |  |  | 2334.088 |
| 1121780 |  |  |  | 2736905 |  | 3897.985 |  |  |  | 35579.58 |
| 446018.9 |  |  |  | 1598166 |  | 3730.176 |  |  |  | 264763.8 |
| 2073694 |  |  |  | 120565.9 |  | 2642.537 |  |  |  | 97149.63 |
| 404775.7 |  |  |  | 5824509 |  | 322158.8 |  |  |  | 107722.4 |
| 525418.1 |  |  |  | 536301.3 |  | 153135.6 |  |  |  | 125349 |
| 450356.2 |  |  |  | 639104.5 |  | 131053.3 |  |  |  | 224207.1 |
| 497284.1 |  |  |  |  |  |  |  |  |  | 126765 |
|  |  |  | D-Mannose | |  | L-Ornithine | |  |  |  |
| D-Mannose | |  | 122330.9 | 72589.05 |  | 25331.99 | 1467.678 |  | L-Proline | |
| 94438.04 | 122330.9 |  | 117472.8 | 164896.7 |  | 24852.56 | 144579 |  | 597682.5 | 773940.6 |
| 125325.4 | 117472.8 |  | 129913.6 | 135243.1 |  | 43168.13 | 119717.1 |  | 1064832 | 645702.5 |
| 136850 | 129913.6 |  | 121589 | 225696.8 |  | 28293.59 | 342457.2 |  | 1738809 | 469183.4 |
| 144680.4 | 121589 |  | 157828.5 | 187562.2 |  | 82810.39 | 349354.3 |  | 1460144 | 773049.1 |
| 198782.9 | 157828.5 |  | 294021.4 | 108507.9 |  | 100104.7 | 192092.2 |  | 1770096 | 862372.2 |
| 126179.7 | 294021.4 |  | 88884.17 | 128250.8 |  | 46278.68 | 189904.9 |  | 1264739 | 549511.9 |
| 125297.7 | 88884.17 |  | 92358.26 | 140167.4 |  | 145145.2 | 1313849 |  | 1954252 | 653315.6 |
| 156932.3 | 92358.26 |  | 80108.56 | 85655.85 |  | 235720.3 | 356172.4 |  | 562672.8 | 945426.7 |
| 131548.2 | 80108.56 |  | 175714.1 | 160408.4 |  | 64550.68 | 848.7398 |  | 1454586 | 719146.4 |
| 127572.5 | 175714.1 |  | 161530.6 | 187370.1 |  | 211142 | 47462.69 |  | 367916.8 | 471592.7 |
| 105615.1 | 161530.6 |  | 109192.1 | 122496.1 |  | 97230.79 | 16707.6 |  | 1350615 | 242932.9 |
| 114821.4 | 109192.1 |  | 115026.3 | 137113.7 |  | 14196.32 | 59573.81 |  | 690303.2 | 574804.2 |
| 131911.8 | 115026.3 |  | 41498.63 | 148333.6 |  | 54521.05 | 93745.41 |  | 664469.9 | 234182.7 |
| 113476.2 | 41498.63 |  | 518.8775 | 134473.1 |  | 44574.96 | 331589.4 |  | 636602.6 | 327401.5 |
| 158624.4 | 518.8775 |  | 120321.6 | 147030.2 |  | 28897.54 | 330858.4 |  | 852799.1 | 200400 |
| 143868.2 | 120321.6 |  | 147614.6 | 62400.09 |  | 695.0322 | 163270.6 |  | 785586.2 | 430288.2 |
| 142510.7 | 147614.6 |  | 138280.9 | 139709.2 |  | 94181.9 | 146218.3 |  | 629716.3 | 592488.3 |
| 133889.8 | 138280.9 |  | 109955.1 | 137324.1 |  | 44486.47 | 111412.8 |  | 594943.4 | 581616.5 |
| 46427.17 | 109955.1 |  | 152048.6 | 152004.9 |  | 273457.1 | 235539 |  | 663486.6 | 553432.5 |
| 78595.9 | 152048.6 |  | 136095.8 | 122670.8 |  | 163950.1 | 649260 |  | 955101 | 957616.5 |
| 101465.2 | 136095.8 |  | 177549.3 | 121745.3 |  | 97772.91 | 455770.9 |  | 1141812 | 956973.7 |
| 93398.35 | 177549.3 |  | 100123.6 | 23305.69 |  | 568781.1 | 203466.3 |  | 1018490 | 805185.4 |
| 122646 | 100123.6 |  | 128063.8 | 149880.5 |  | 59313.81 | 1151872 |  | 603631 | 505403.4 |
| 137543.3 | 128063.8 |  | 95268.94 | 148358.4 |  | 91293.5 | 652030 |  | 932219.2 | 635941.3 |
| 138889.6 | 95268.94 |  | 95928.38 | 224283.8 |  | 59530.4 | 442520.6 |  | 1432130 | 539171.9 |
| 156712.2 | 95928.38 |  | 90778.12 | 190165.1 |  | 216617.3 | 266806.9 |  | 1818119 | 647611.1 |
| 125655.7 | 90778.12 |  | 68382.61 | 90773.55 |  | 1210.378 | 320402 |  | 3768370 | 790445 |
| 116032.7 | 68382.61 |  | 104184.3 | 110839.1 |  | 241485.7 | 242142.7 |  | 6641716 | 481105.4 |
| 117193 | 104184.3 |  | 135617.7 | 115426.8 |  | 119636.4 | 269937.4 |  | 1483987 | 818228.7 |
| 105938.2 | 135617.7 |  | 148.6388 | 160785.7 |  | 85238.62 | 569766.6 |  | 1617857 | 891609.4 |
| 100883.7 | 148.6388 |  | 141.091 | 141378.4 |  | 146087.5 | 442336.7 |  | 2346606 | 740522 |
| 103631.1 | 141.091 |  | 485.1469 | 96470.09 |  | 22367.62 | 0 |  | 2838206 | 766045.5 |
| 93189.5 | 485.1469 |  | 0 | 118036.7 |  | 63078.38 | 195419 |  | 906289.6 | 727636.3 |
| 116561.6 | 0 |  | 127165 | 125541.6 |  | 300248.4 | 215155.7 |  | 1015910 | 781571.6 |
| 95000.08 | 127165 |  | 1914.452 | 153163.2 |  | 409959.9 | 109192.3 |  | 799532.5 | 722104.9 |
| 113812 | 1914.452 |  | 0 | 135758.9 |  | 147088.6 | 86063.69 |  | 570015.5 | 1090961 |
| 123585.1 | 0 |  | 60734.15 | 127768.6 |  | 140050.7 | 31059.78 |  | 1034664 | 107379.7 |
| 118477.2 | 60734.15 |  | 38927.26 | 157174 |  | 0 | 7289.294 |  | 882323.1 | 134458.5 |
| 95206.93 | 38927.26 |  | 4736.7 | 149008.6 |  | 765724.3 |  |  | 331811.3 | 22025.28 |
| 121650 | 4736.7 |  | 151857.4 | 126867.2 |  | 298538.2 |  |  |  | 233983.8 |
| 110867.3 | 151857.4 |  | 280081.3 | 97989.88 |  | 575.1936 |  |  |  | 1181723 |
| 107295.5 | 280081.3 |  | 133035.3 | 133482 |  | 622448.1 |  |  |  | 401771.8 |
| 123590.7 | 133035.3 |  |  | 91754.39 |  | 71364.51 |  |  |  | 92930.63 |
| 118728.7 |  |  |  | 200673.3 |  | 731.3204 |  |  |  | 995981 |
| 141837.9 |  |  |  | 191204 |  | 32549.71 |  |  |  | 743057.5 |
| 145228.5 |  |  |  | 140370.9 |  | 5552.523 |  |  |  | 1084342 |
| 96648.31 |  |  |  | 130948.6 |  | 0 |  |  |  | 292065.9 |
| 125638.5 |  |  |  | 157805.9 |  | 2231.751 |  |  |  | 520171.4 |
| 138240.3 |  |  |  | 218497.3 |  | 0 |  |  |  | 758056.4 |
| 123864.2 |  |  |  | 215363.4 |  | 288052.5 |  |  |  | 788541.7 |
|  |  |  |  | 169142.2 |  | 274513.8 |  |  |  |  |
| Glycine | |  |  | 151979.3 |  | 100666.4 |  |  | L-Ornithine | |
| 539639.1 | 716268 |  |  | 151062.6 |  | 301548.9 |  |  | 1467.678 | 64036.08 |
| 1417048 | 1080786 |  |  | 115812.2 |  | 140453.2 |  |  | 144579 | 12826.1 |
| 1159071 | 1109667 |  |  | 184994.5 |  | 540460.9 |  |  | 119717.1 | 16792.27 |
| 795546.8 | 1440344 |  |  | 200789.3 |  | 308614.2 |  |  | 342457.2 | 43637.02 |
| 1234044 | 1275471 |  |  | 173924 |  | 690.4299 |  |  | 349354.3 | 78743.65 |
| 1283633 | 1077647 |  |  | 128870.2 |  | 12628.23 |  |  | 192092.2 | 8944.061 |
| 1103320 | 1156091 |  |  | 226168 |  | 0 |  |  | 189904.9 | 20339.73 |
| 1529256 | 1638338 |  |  | 129876.7 |  | 322.123 |  |  | 1313849 | 74679.17 |
| 1399900 | 2200469 |  |  | 193393.1 |  | 255.3698 |  |  | 356172.4 | 26333.75 |
| 1041577 | 1485472 |  |  | 135785.7 |  | 0 |  |  | 848.7398 | 16322.87 |
| 510324 | 2903771 |  |  | 146314 |  | 404.3482 |  |  | 47462.69 | 2277.706 |
| 1040456 | 1409233 |  |  | 245498.7 |  | 447677.3 |  |  | 16707.6 | 77387.27 |
| 1068193 | 1292286 |  |  | 143711.6 |  | 36878.94 |  |  | 59573.81 | 4313.775 |
| 767936.8 | 2142585 |  |  | 168634.7 |  | 16690.39 |  |  | 93745.41 | 667.7185 |
| 962841.9 | 2477647 |  |  |  |  |  |  |  | 331589.4 | 4535.563 |
| 1307210 | 939964.4 |  | Glycine | |  | L-Serine | |  | 330858.4 | 51688.82 |
| 1807618 | 1699093 |  | 716268 | 661937.9 |  | 127355.8 | 416231 |  | 163270.6 | 133204 |
| 1568369 | 1669954 |  | 1080786 | 1099860 |  | 172898.8 | 395437.8 |  | 146218.3 | 141529.1 |
| 1363876 | 1129020 |  | 1109667 | 751658.4 |  | 202103.8 | 338678.4 |  | 111412.8 | 183200.5 |
| 1884778 | 1248887 |  | 1440344 | 1101080 |  | 290793.4 | 444492.9 |  | 235539 | 355550.7 |
| 1794266 | 1490251 |  | 1275471 | 1636469 |  | 336450 | 634621.8 |  | 649260 | 311043.8 |
| 1881437 | 1029890 |  | 1077647 | 430157.2 |  | 483170.5 | 304037.2 |  | 455770.9 | 356512 |
| 983898.6 | 1406125 |  | 1156091 | 833964.8 |  | 131098.7 | 348733.1 |  | 203466.3 | 120905.4 |
| 1599906 | 1789552 |  | 1638338 | 658793.5 |  | 170075.5 | 547767.4 |  | 1151872 | 3459.994 |
| 1299499 | 1124697 |  | 2200469 | 621861 |  | 86674.4 | 486845.6 |  | 652030 | 41942.88 |
| 1438814 | 2623585 |  | 1485472 | 1335474 |  | 179014.3 | 536728.2 |  | 442520.6 | 61177.4 |
| 1487562 | 978159.9 |  | 2903771 | 1257203 |  | 763728 | 149325.4 |  | 266806.9 | 111207.6 |
| 1264709 | 1864214 |  | 1409233 | 1011109 |  | 593167.8 | 424603.4 |  | 320402 | 44420.11 |
| 1932221 | 2054732 |  | 1292286 | 677134.8 |  | 481449.7 | 421301.8 |  | 242142.7 | 105627.4 |
| 1669629 | 2243221 |  | 2142585 | 1504583 |  | 545178.8 | 548571.5 |  | 269937.4 | 180541.4 |
| 1775162 | 3284177 |  | 2477647 | 1608569 |  | 558878.6 | 911857.7 |  | 569766.6 | 9852.788 |
| 1506833 | 2369615 |  | 939964.4 | 752344.9 |  | 92931.71 | 496732.3 |  | 442336.7 | 171521.6 |
| 1567412 | 51120.89 |  | 1699093 | 567612.8 |  | 154565 | 890827.6 |  | 0 | 102169.9 |
| 1157438 | 2354023 |  | 1669954 | 1185752 |  | 529443.3 | 468226.1 |  | 195419 | 272778.3 |
| 1300126 | 2410948 |  | 1129020 | 1030838 |  | 137430.1 | 300454.3 |  | 215155.7 | 77012.76 |
| 1897949 | 2221009 |  | 1248887 | 2129628 |  | 387337.8 | 635294.9 |  | 109192.3 | 256899.1 |
| 4967.218 | 600464.7 |  | 1490251 | 1741678 |  | 87496.96 | 531458.2 |  | 86063.69 | 0 |
| 58601.13 | 955613.3 |  | 1029890 | 1223707 |  | 113690.7 | 900026.3 |  | 31059.78 | 154.5745 |
| 2091.93 | 2674201 |  | 1406125 | 794813.3 |  | 618672 | 565141.5 |  | 7289.294 | 66.97999 |
| 26255.13 | 1467279 |  | 1789552 | 1730215 |  | 330259.2 | 168443.9 |  |  | 0 |
| 1998532 | 1675791 |  | 1124697 | 1936012 |  | 191978 | 212314.4 |  |  | 209689 |
| 706677.8 | 2191552 |  | 2623585 | 1539482 |  | 136466.2 | 386581.6 |  |  | 1772.453 |
| 5445.306 | 2219162 |  | 978159.9 | 2717338 |  | 435292.1 | 277426 |  |  | 471.1032 |
| 1536700 |  |  | 1864214 | 207963.2 |  | 749.313 | 721194.4 |  |  | 42190.32 |
| 1704987 |  |  | 2054732 | 1917096 |  | 255890.1 | 268758.8 |  |  | 326603.9 |
| 1932659 |  |  | 2243221 | 2439561 |  | 277796.1 | 252932.3 |  |  | 336803.9 |
| 1019483 |  |  | 3284177 | 1796783 |  | 142609.5 | 773580.1 |  |  | 36945.35 |
| 788550.3 |  |  | 2369615 | 2680345 |  | 286454.9 | 464729.7 |  |  | 10434.03 |
| 1482511 |  |  | 51120.89 | 1539082 |  | 194375.3 | 3894.257 |  |  | 55458.97 |
| 1399231 |  |  | 2354023 | 1047092 |  | 82753.79 | 769404.6 |  |  | 45923.45 |
|  |  |  | 2410948 | 2945594 |  | 164465.6 | 410798.7 |  |  |  |
| L-Alanine | |  | 2221009 | 2686016 |  | 466528.7 | 186250.8 |  | L-Serine | |
| 226931.1 | 294947.9 |  | 600464.7 | 2244668 |  | 240627.3 | 192873 |  | 416231 | 111510.6 |
| 91290.62 | 159907.3 |  | 955613.3 | 2150105 |  | 282417.6 | 236190.2 |  | 395437.8 | 81613.35 |
| 142004.7 | 45706.56 |  | 2674201 | 78386.99 |  | 2079.828 | 176520.4 |  | 338678.4 | 70253.61 |
| 183672.2 | 120031.1 |  | 1467279 | 3077735 |  | 1024309 |  |  | 444492.9 | 149828.7 |
| 175113.2 | 99836.39 |  | 1675791 | 1952094 |  | 244402.8 |  |  | 634621.8 | 327236.7 |
| 106236 | 66472.45 |  | 2191552 | 154512.6 |  | 3177.728 |  |  | 304037.2 | 228595.2 |
| 99510 | 209053.3 |  | 2219162 | 2220619 |  | 260875.9 |  |  | 348733.1 | 217905.1 |
| 121075.4 | 147287.4 |  |  | 1385862 |  | 92122.24 |  |  | 547767.4 | 437031.9 |
| 103193.8 | 311793.8 |  |  | 149823.4 |  | 2740.609 |  |  | 486845.6 | 187591.5 |
| 178224.9 | 255148.4 |  |  | 1351401 |  | 4926.614 |  |  | 536728.2 | 5475.657 |
| 103727.5 | 304324.1 |  |  | 1314014 |  | 32374.19 |  |  | 149325.4 | 234405.7 |
| 199097.7 | 164971.2 |  |  | 88737.37 |  | 2128.547 |  |  | 424603.4 | 504297 |
| 130135.8 | 187339.2 |  |  | 1461249 |  | 18793.79 |  |  | 421301.8 | 151078.3 |
| 139982.5 | 277096.5 |  |  | 19648.65 |  | 4582.981 |  |  | 548571.5 | 268544.6 |
| 138618.3 | 272985.1 |  |  | 1911907 |  | 219477.8 |  |  | 911857.7 | 56210.85 |
| 232504.2 | 237637.5 |  |  | 2740564 |  | 213324.5 |  |  | 496732.3 | 123432.9 |
| 293291.2 | 282320.6 |  |  | 1249145 |  | 65597.03 |  |  | 890827.6 | 802494.6 |
| 270365.5 | 155731.6 |  |  | 2599051 |  | 388865.4 |  |  | 468226.1 | 484562.3 |
| 152440.8 | 174905.6 |  |  | 1569160 |  | 56011.71 |  |  | 300454.3 | 228563.4 |
| 235920.1 | 153067.5 |  |  | 2999527 |  | 452914.1 |  |  | 635294.9 | 599546.5 |
| 224076.4 | 32144.31 |  |  | 10299533 |  | 130068.7 |  |  | 531458.2 | 169083.6 |
| 297467.8 | 1195.454 |  |  | 978.4001 |  | 58.6182 |  |  | 900026.3 | 419439 |
| 235879.3 | 7375.611 |  |  | 77504.77 |  | 6469.239 |  |  | 565141.5 | 593323.1 |
| 20881.17 | 35942.72 |  |  | 58.55713 |  | 0 |  |  | 168443.9 | 176141.3 |
| 1259.674 | 161218 |  |  | 28239.05 |  | 0 |  |  | 212314.4 | 107672.9 |
| 132065.4 | 167180.1 |  |  | 5997.481 |  | 5264.188 |  |  | 386581.6 | 191217.4 |
| 26741.64 | 84706.66 |  |  | 5139.614 |  | 3195.348 |  |  | 277426 | 222689.4 |
| 17105.62 | 79763.21 |  |  | 768.7986 |  | 0 |  |  | 721194.4 | 118760.5 |
| 2279.932 | 161749.8 |  |  | 1886181 |  | 637229.7 |  |  | 268758.8 | 224882.4 |
| 99910.61 | 72266.23 |  |  | 1251152 |  | 88113.11 |  |  | 252932.3 | 195182.9 |
| 101511.8 | 382774.5 |  |  | 1498172 |  | 141061 |  |  | 773580.1 | 154816.4 |
| 102664.8 | 138052.5 |  |  |  |  |  |  |  | 464729.7 | 170998.4 |
| 113503.1 | 3916.479 |  | L-Alanine | |  | Cystathionine | |  | 3894.257 | 161744.3 |
| 250717.7 | 200960.1 |  | 294947.9 | 182821.9 |  | 7195.762 | 37534.21 |  | 769404.6 | 121625 |
| 81800.64 | 171307.2 |  | 159907.3 | 137251.8 |  | 24147.58 | 50417.28 |  | 410798.7 | 93166.21 |
| 119218.4 | 27240.1 |  | 45706.56 | 110068.8 |  | 44172.51 | 36109.14 |  | 186250.8 | 290548.1 |
| 2391.582 | 4381.261 |  | 120031.1 | 65784.13 |  | 50886.87 | 51986.38 |  | 192873 | 252.1462 |
| 2047.638 | 56808.45 |  | 99836.39 | 150692.2 |  | 31605.74 | 122101.4 |  | 236190.2 | 3664.445 |
| 2971.244 | 268594.2 |  | 66472.45 | 123284.2 |  | 36314.83 | 29339.97 |  | 176520.4 | 0 |
| 2410.25 | 267866.6 |  | 209053.3 | 105679.1 |  | 46401.31 | 7462.334 |  |  | 8623.97 |
| 103780.6 | 323988.8 |  | 147287.4 | 110394.8 |  | 39968.32 | 33513.98 |  |  | 145694.7 |
| 2286.206 | 214697.9 |  | 311793.8 | 85395.52 |  | 41259.85 | 24714.76 |  |  | 6476.225 |
| 2334.088 | 124392.9 |  | 255148.4 | 180174.7 |  | 22540.32 | 20209.13 |  |  | 1322.278 |
| 35579.58 |  |  | 304324.1 | 131057.9 |  | 29567.09 | 1385.35 |  |  | 61859.64 |
| 264763.8 |  |  | 164971.2 | 131458.8 |  | 22870.97 | 41137.96 |  |  | 918915.4 |
| 97149.63 |  |  | 187339.2 | 165322.4 |  | 28781.74 | 36051.32 |  |  | 32802.09 |
| 107722.4 |  |  | 277096.5 | 173319.8 |  | 26624.05 | 38772.22 |  |  | 267093.9 |
| 125349 |  |  | 272985.1 | 161294.2 |  | 21846.87 | 39593.38 |  |  | 23684.87 |
| 224207.1 |  |  | 237637.5 | 186369.1 |  | 27356.78 | 52495.5 |  |  | 406382.9 |
| 126765 |  |  | 282320.6 | 76839.53 |  | 26428.19 | 46858.1 |  |  | 382072.8 |
|  |  |  | 155731.6 | 208653.2 |  | 38634.84 | 42219.63 |  |  |  |
| L-Proline | |  | 174905.6 | 183929.9 |  | 36443.68 | 70692.18 |  | L-Tyrosine | |
| 773940.6 | 708854.4 |  | 153067.5 | 217022 |  | 41610.72 | 43657.12 |  | 734110.1 | 252790.2 |
| 645702.5 | 599393.2 |  | 32144.31 | 198458 |  | 12309.38 | 60282.31 |  | 1940420 | 244557.7 |
| 469183.4 | 513984.5 |  | 1195.454 | 108346.4 |  | 20186.94 | 69741.1 |  | 841408.7 | 216690.2 |
| 773049.1 | 722488.7 |  | 7375.611 | 219658.7 |  | 34888.6 | 47520.57 |  | 734893.5 | 365047.8 |
| 862372.2 | 786133.8 |  | 35942.72 | 120231.2 |  | 1481.863 | 13380.9 |  | 368614.1 | 278138.5 |
| 549511.9 | 455821 |  | 161218 | 73213.46 |  | 24016.42 | 51177.5 |  | 580560.7 | 175698.5 |
| 653315.6 | 462427.7 |  | 167180.1 | 1154.31 |  | 24056.98 | 67763.74 |  | 791620.9 | 243700.3 |
| 945426.7 | 779829.9 |  | 84706.66 | 82273.67 |  | 30002.53 | 61473.99 |  | 354714.4 | 271606 |
| 719146.4 | 1015240 |  | 79763.21 | 18491.16 |  | 0 | 37205.67 |  | 686798.8 | 227811.1 |
| 471592.7 | 493576.8 |  | 161749.8 | 139013.4 |  | 17849.85 | 77837.59 |  | 188088.5 | 203815.4 |
| 242932.9 | 1106815 |  | 72266.23 | 79408.45 |  | 34928.99 | 55904.5 |  | 301773.2 | 136946.5 |
| 574804.2 | 650278.5 |  | 382774.5 | 45286.37 |  | 37949.47 | 100951 |  | 623302.5 | 243679.9 |
| 234182.7 | 733482.8 |  | 138052.5 | 226365.3 |  | 37670.72 | 64820.88 |  | 396994.4 | 229113.2 |
| 327401.5 | 1469579 |  | 3916.479 | 86527.58 |  | 14294.94 | 0 |  | 255612.3 | 181777.1 |
| 200400 | 1802207 |  | 200960.1 | 204068.3 |  | 18891.26 | 47026.53 |  | 315851.7 | 308647.5 |
| 430288.2 | 699351 |  | 171307.2 | 31527.94 |  | 8506.445 | 58757.14 |  | 311916.5 | 317918.8 |
| 592488.3 | 1542703 |  | 27240.1 | 215164.6 |  | 34499.76 | 65144.16 |  | 380943.5 | 159776.7 |
| 581616.5 | 881078.1 |  | 4381.261 | 190945.1 |  | 6804.169 | 48351.86 |  | 324033.9 | 260749.3 |
| 553432.5 | 577595.1 |  | 56808.45 | 155275.4 |  | 22392.07 | 23085.2 |  | 194069.9 | 171796.1 |
| 957616.5 | 672946.5 |  | 268594.2 | 1928.188 |  | 0 | 64774.7 |  | 171812.5 | 194430.5 |
| 956973.7 | 768970 |  | 267866.6 | 188847.9 |  | 60170.39 |  |  | 230004.4 | 397490.5 |
| 805185.4 | 1319611 |  | 323988.8 | 111624 |  | 3003.575 |  |  | 417943.3 | 244337.8 |
| 505403.4 | 620399 |  | 214697.9 | 60952.31 |  | 0 |  |  | 348204.3 | 220676 |
| 635941.3 | 544171.9 |  | 124392.9 | 118745 |  | 15684.31 |  |  | 244837.1 | 210084.3 |
| 539171.9 | 1180409 |  |  | 99360.95 |  | 3671.987 |  |  | 206264.7 | 268962.2 |
| 647611.1 | 1449563 |  |  | 2405.85 |  | 63.79436 |  |  | 176414.7 | 363053.5 |
| 790445 | 761750.7 |  |  | 2164.448 |  | 0 |  |  | 268089.7 | 191216.4 |
| 481105.4 | 1323616 |  |  | 1314.12 |  | 2194.129 |  |  | 106512.6 | 165622.2 |
| 818228.7 | 1415939 |  |  | 1822.906 |  | 0 |  |  | 320929.4 | 208109.9 |
| 891609.4 | 1206680 |  |  | 2446.194 |  | 1634.623 |  |  | 275138.7 | 178396.1 |
| 740522 | 2796684 |  |  | 1782.823 |  | 0 |  |  | 249901.7 | 280969 |
| 766045.5 | 3049016 |  |  | 248374.3 |  | 32605.07 |  |  | 208730.8 | 300277.2 |
| 727636.3 | 335615.1 |  |  | 174096.8 |  | 39026.91 |  |  | 766126.2 | 174321.6 |
| 781571.6 | 2407331 |  |  | 27295.66 |  | 930.8355 |  |  | 440361.8 | 221850.2 |
| 722104.9 | 2055156 |  |  | 273141.9 |  | 62431.41 |  |  | 366684.3 | 163783.2 |
| 1090961 | 1925809 |  |  | 109403.3 |  | 1400.698 |  |  | 365400.6 | 277675.8 |
| 107379.7 | 1602933 |  |  | 195066.2 |  | 28486.33 |  |  | 344000.2 | 120726.7 |
| 134458.5 | 2427998 |  |  | 57156.01 |  | 26508.16 |  |  | 580737.7 | 165427.1 |
| 22025.28 | 2944120 |  |  | 6448.67 |  | 0 |  |  | 339402.2 | 96202.22 |
| 233983.8 | 811206.8 |  |  | 2137.993 |  | 0 |  |  |  | 261142.1 |
| 1181723 | 1051923 |  |  | 4492.577 |  | 0 |  |  |  | 230640.6 |
| 401771.8 | 1103778 |  |  | 3577.81 |  | 0 |  |  |  | 341192.3 |
| 92930.63 | 986746.9 |  |  | 3897.985 |  | 0 |  |  |  | 116920.5 |
| 995981 |  |  |  | 3730.176 |  | 0 |  |  |  | 190178 |
| 743057.5 |  |  |  | 2642.537 |  | 0 |  |  |  | 166348.8 |
| 1084342 |  |  |  | 322158.8 |  | 61698.01 |  |  |  | 155480.5 |
| 292065.9 |  |  |  | 153135.6 |  | 45932.78 |  |  |  | 165797.2 |
| 520171.4 |  |  |  | 131053.3 |  | 28242.93 |  |  |  | 247289.8 |
| 758056.4 |  |  |  |  |  |  |  |  |  | 216343.2 |
| 788541.7 |  |  | L-Proline | |  | Creatine | |  |  | 205346.6 |
|  |  |  | 708854.4 | 305523.8 |  | 38251.82 | 49326.35 |  |  |  |
| L-Ornithine | |  | 599393.2 | 815112.7 |  | 40752.1 | 37675.09 |  | Cystathionine | |
| 64036.08 | 80396.51 |  | 513984.5 | 611067.5 |  | 50969.83 | 71280.27 |  | 37534.21 | 22286.9 |
| 12826.1 | 30081.7 |  | 722488.7 | 963890.3 |  | 38792.4 | 65771.71 |  | 50417.28 | 13495.72 |
| 16792.27 | 75201.15 |  | 786133.8 | 847030.7 |  | 54561.31 | 187468.9 |  | 36109.14 | 42132.7 |
| 43637.02 | 73424.2 |  | 455821 | 517205.7 |  | 48753.36 | 229203 |  | 51986.38 | 57861.64 |
| 78743.65 | 30330.79 |  | 462427.7 | 451399.9 |  | 42928.23 | 86242.34 |  | 122101.4 | 72645.18 |
| 8944.061 | 11713.53 |  | 779829.9 | 419452.9 |  | 42349.26 | 33324.2 |  | 29339.97 | 41399.99 |
| 20339.73 | 5820.097 |  | 1015240 | 335392.2 |  | 34157.43 | 89056.39 |  | 7462.334 | 30498.67 |
| 74679.17 | 46279.42 |  | 493576.8 | 461595.6 |  | 37248.97 | 110014.2 |  | 33513.98 | 55348.36 |
| 26333.75 | 74930.65 |  | 1106815 | 467152.8 |  | 459.0349 | 169437.8 |  | 24714.76 | 31692.28 |
| 16322.87 | 33558.69 |  | 650278.5 | 676282.6 |  | 94610 | 49569.27 |  | 20209.13 | 17175.35 |
| 2277.706 | 232059.4 |  | 733482.8 | 487997.3 |  | 59255.97 | 84304.55 |  | 1385.35 | 13342.88 |
| 77387.27 | 80026.79 |  | 1469579 | 621547.8 |  | 51940.15 | 62521.83 |  | 41137.96 | 20419.4 |
| 4313.775 | 162463 |  | 1802207 | 636903.9 |  | 53960.16 | 40938.93 |  | 36051.32 | 18770.02 |
| 667.7185 | 288070.9 |  | 699351 | 561075.4 |  | 70249.11 | 94871.93 |  | 38772.22 | 27025.1 |
| 4535.563 | 284188.1 |  | 1542703 | 157311.9 |  | 39321.65 | 101266.9 |  | 39593.38 | 12879.14 |
| 51688.82 | 168481.7 |  | 881078.1 | 777076 |  | 72042.87 | 77247.56 |  | 52495.5 | 26703.37 |
| 133204 | 281476 |  | 577595.1 | 582187.1 |  | 91157.51 | 76023.86 |  | 46858.1 | 38162.14 |
| 141529.1 | 110597.8 |  | 672946.5 | 1071340 |  | 49601.51 | 74795.21 |  | 42219.63 | 40338.3 |
| 183200.5 | 80055.88 |  | 768970 | 744719.1 |  | 58378.66 | 51351.02 |  | 70692.18 | 13624.21 |
| 355550.7 | 86162.78 |  | 1319611 | 600401.5 |  | 53347.75 | 77709.38 |  | 43657.12 | 49536.7 |
| 311043.8 | 32318.08 |  | 620399 | 903743.6 |  | 63008.11 | 73114.39 |  | 60282.31 | 13224.81 |
| 356512 | 716422 |  | 544171.9 | 664702.8 |  | 24992.7 | 26884.61 |  | 69741.1 | 22711.47 |
| 120905.4 | 50813.91 |  | 1180409 | 1286325 |  | 18237.94 | 30741.85 |  | 47520.57 | 27080.34 |
| 3459.994 | 27075.79 |  | 1449563 | 735920.9 |  | 1335.228 | 2659.929 |  | 13380.9 | 38113.63 |
| 41942.88 | 164611 |  | 761750.7 | 1178407 |  | 881.1732 | 36752.99 |  | 51177.5 | 10553.99 |
| 61177.4 | 644466.7 |  | 1323616 | 4422.451 |  | 396.36 | 26086.69 |  | 67763.74 | 4981.376 |
| 111207.6 | 46517.74 |  | 1415939 | 912542.3 |  | 1660.68 | 0 |  | 61473.99 | 12139.76 |
| 44420.11 | 202782.9 |  | 1206680 | 1411778 |  | 282.5176 | 63610.43 |  | 37205.67 | 11711.95 |
| 105627.4 | 129117.3 |  | 2796684 | 1011131 |  | 1096.383 | 0 |  | 77837.59 | 14360.53 |
| 180541.4 | 560294.6 |  | 3049016 | 1212368 |  | 26553.18 | 34212.54 |  | 55904.5 | 17772.52 |
| 9852.788 | 557472.4 |  | 335615.1 | 1099732 |  | 1086.68 | 1872.971 |  | 100951 | 24243.26 |
| 171521.6 | 210689.9 |  | 2407331 | 630252.2 |  | 65433.68 | 60544.72 |  | 64820.88 | 18964.84 |
| 102169.9 | 0 |  | 2055156 | 1554364 |  | 1737.188 | 80869.61 |  | 0 | 8271.963 |
| 272778.3 | 263033.9 |  | 1925809 | 1215186 |  | 65616.31 | 112871.6 |  | 47026.53 | 21688.85 |
| 77012.76 | 338735.8 |  | 1602933 | 940319.4 |  | 1397.16 | 69197 |  | 58757.14 | 4875.646 |
| 256899.1 | 227534.2 |  | 2427998 | 1183677 |  | 1257.263 | 47982.38 |  | 65144.16 | 22664.51 |
| 0 | 97691.41 |  | 2944120 | 362889.7 |  | 2108.547 | 93781.39 |  | 48351.86 | 0 |
| 154.5745 | 266277.2 |  | 811206.8 | 2309309 |  | 22779.67 |  |  | 23085.2 | 0 |
| 66.97999 | 545091.1 |  | 1051923 | 1947940 |  | 1160.946 |  |  | 64774.7 | 0 |
| 0 | 253180.2 |  | 1103778 | 147412.7 |  | 563.73 |  |  |  | 0 |
| 209689 | 286103.7 |  | 986746.9 | 770978.3 |  | 705.8771 |  |  |  | 13876.34 |
| 1772.453 | 45760.81 |  |  | 422968.1 |  | 54308.97 |  |  |  | 0 |
| 471.1032 | 662380.6 |  |  | 266792.4 |  | 1145.204 |  |  |  | 0 |
| 42190.32 |  |  |  | 822479 |  | 1495.615 |  |  |  | 3430.524 |
| 326603.9 |  |  |  | 1085724 |  | 1568.791 |  |  |  | 36797.62 |
| 336803.9 |  |  |  | 397597.6 |  | 1223.559 |  |  |  | 27793.82 |
| 36945.35 |  |  |  | 779385.9 |  | 1057.803 |  |  |  | 49063.23 |
| 10434.03 |  |  |  | 185407.6 |  | 1097.899 |  |  |  | 25658.63 |
| 55458.97 |  |  |  | 1181672 |  | 23645.6 |  |  |  | 61055.24 |
| 45923.45 |  |  |  | 1130425 |  | 109861.6 |  |  |  | 44704.19 |
|  |  |  |  | 1788983 |  | 455.6668 |  |  |  |  |
| L-Serine | |  |  | 1902447 |  | 123900.7 |  |  | Creatine | |
| 111510.6 | 443077.2 |  |  | 705238.8 |  | 62925.45 |  |  | 49326.35 | 1065.411 |
| 81613.35 | 159336.6 |  |  | 2302136 |  | 90924.27 |  |  | 37675.09 | 47615.26 |
| 70253.61 | 184777.1 |  |  | 2082900 |  | 51900.99 |  |  | 71280.27 | 38160.2 |
| 149828.7 | 150526.9 |  |  | 28023.27 |  | 3301.179 |  |  | 65771.71 | 57522.83 |
| 327236.7 | 235384.9 |  |  | 657018.9 |  | 992.7598 |  |  | 187468.9 | 61007.89 |
| 228595.2 | 18485.27 |  |  | 404.942 |  | 1061.69 |  |  | 229203 | 45274.69 |
| 217905.1 | 652772.8 |  |  | 34278.01 |  | 1168.181 |  |  | 86242.34 | 37230.13 |
| 437031.9 | 900387.2 |  |  | 1194762 |  | 1683.19 |  |  | 33324.2 | 64378.85 |
| 187591.5 | 792970.8 |  |  | 800392.4 |  | 2388.032 |  |  | 89056.39 | 52190.22 |
| 5475.657 | 140986.5 |  |  | 56804.37 |  | 2433.145 |  |  | 110014.2 | 62308 |
| 234405.7 | 1657142 |  |  | 1964614 |  | 54345.53 |  |  | 169437.8 | 44389.45 |
| 504297 | 435074.6 |  |  | 752835.3 |  | 52460.34 |  |  | 49569.27 | 38809.48 |
| 151078.3 | 513070.8 |  |  | 854003.9 |  | 34075.38 |  |  | 84304.55 | 55367.46 |
| 268544.6 | 903656.2 |  |  |  |  |  |  |  | 62521.83 | 43502.27 |
| 56210.85 | 265621.7 |  | L-Ornithine | |  | Palmitic acid | |  | 40938.93 | 49027.91 |
| 123432.9 | 171141.1 |  | 80396.51 | 25331.99 |  | 954176.6 | 127139 |  | 94871.93 | 61096.38 |
| 802494.6 | 218171 |  | 30081.7 | 24852.56 |  | 1058206 | 840691.8 |  | 101266.9 | 118547.2 |
| 484562.3 | 163481.4 |  | 75201.15 | 43168.13 |  | 784939.5 | 1425486 |  | 77247.56 | 156044.4 |
| 228563.4 | 314555.8 |  | 73424.2 | 28293.59 |  | 1841482 | 324597.5 |  | 76023.86 | 73777.06 |
| 599546.5 | 263636.8 |  | 30330.79 | 82810.39 |  | 963980.6 | 778897.7 |  | 74795.21 | 117102.4 |
| 169083.6 | 213049.6 |  | 11713.53 | 100104.7 |  | 878557.9 | 1596994 |  | 51351.02 | 33196.52 |
| 419439 | 500107.8 |  | 5820.097 | 46278.68 |  | 384235.7 | 864826.5 |  | 77709.38 | 55818.6 |
| 593323.1 | 119143.1 |  | 46279.42 | 145145.2 |  | 605243.5 | 649488 |  | 73114.39 | 62771.33 |
| 176141.3 | 221432.6 |  | 74930.65 | 235720.3 |  | 341774 | 427907.9 |  | 26884.61 | 22536.83 |
| 107672.9 | 261097.3 |  | 33558.69 | 64550.68 |  | 1212022 | 1046602 |  | 30741.85 | 35240.6 |
| 191217.4 | 371153.1 |  | 232059.4 | 211142 |  | 825181.3 | 1084853 |  | 2659.929 | 0 |
| 222689.4 | 68330.78 |  | 80026.79 | 97230.79 |  | 549900.4 | 913991.3 |  | 36752.99 | 26026.42 |
| 118760.5 | 338364.8 |  | 162463 | 14196.32 |  | 644968 | 470650.3 |  | 26086.69 | 737.4663 |
| 224882.4 | 234060 |  | 288070.9 | 54521.05 |  | 971845.8 | 288606.7 |  | 0 | 27568.28 |
| 195182.9 | 199614.6 |  | 284188.1 | 44574.96 |  | 540449 | 283549.4 |  | 63610.43 | 16827.62 |
| 154816.4 | 666512.4 |  | 168481.7 | 28897.54 |  | 708358.7 | 234840 |  | 0 | 268.7651 |
| 170998.4 | 93683.41 |  | 281476 | 695.0322 |  | 338938 | 687599.7 |  | 34212.54 | 32499.26 |
| 161744.3 | 1972.345 |  | 110597.8 | 94181.9 |  | 1206103 | 135239.4 |  | 1872.971 | 25125.39 |
| 121625 | 119001.1 |  | 80055.88 | 44486.47 |  | 762911.5 | 237983.3 |  | 60544.72 | 51186.82 |
| 93166.21 | 30092.07 |  | 86162.78 | 273457.1 |  | 103386.3 | 681730.6 |  | 80869.61 | 596.6619 |
| 290548.1 | 85962.86 |  | 32318.08 | 163950.1 |  | 691448.3 | 201542.8 |  | 112871.6 | 28364.79 |
| 252.1462 | 37084.03 |  | 716422 | 97772.91 |  | 743386.2 | 241673.5 |  | 69197 | 1517.066 |
| 3664.445 | 81228.55 |  | 50813.91 | 568781.1 |  | 293279.9 | 355241.9 |  | 47982.38 | 1007.199 |
| 0 | 278516.9 |  | 27075.79 | 59313.81 |  | 1891353 | 681590.5 |  | 93781.39 | 1485.078 |
| 8623.97 | 748996.4 |  | 164611 | 91293.5 |  | 961090.8 | 1512800 |  |  | 1800.396 |
| 145694.7 | 574267.4 |  | 644466.7 | 59530.4 |  | 595423 | 413402.2 |  |  | 786.6787 |
| 6476.225 | 234341 |  | 46517.74 | 216617.3 |  | 585423.6 | 367120.8 |  |  | 1616.437 |
| 1322.278 | 165090.8 |  | 202782.9 | 1210.378 |  | 953696.2 | 238640.2 |  |  | 2192.579 |
| 61859.64 |  |  | 129117.3 | 241485.7 |  | 1002574 | 470219.5 |  |  | 680.7938 |
| 918915.4 |  |  | 560294.6 | 119636.4 |  | 174822.1 | 336552.4 |  |  | 90155.85 |
| 32802.09 |  |  | 557472.4 | 85238.62 |  | 953046.3 | 543352.5 |  |  | 600.5712 |
| 267093.9 |  |  | 210689.9 | 146087.5 |  | 1222195 | 482524.3 |  |  | 70259.2 |
| 23684.87 |  |  | 0 | 22367.62 |  | 655415.3 | 494875.3 |  |  | 46162.58 |
| 406382.9 |  |  | 263033.9 | 63078.38 |  | 742424.7 | 186289.8 |  |  | 52093.44 |
| 382072.8 |  |  | 338735.8 | 300248.4 |  | 850168.6 | 218532.5 |  |  | 59664.32 |
|  |  |  | 227534.2 | 409959.9 |  | 434195.3 | 599158.8 |  |  |  |
| L-Tyrosine | |  | 97691.41 | 147088.6 |  | 336065.1 | 486680 |  | Palmitic acid | |
| 252790.2 | 375317 |  | 266277.2 | 140050.7 |  | 385518.7 | 1038040 |  | 127139 | 539558.4 |
| 244557.7 | 323298.3 |  | 545091.1 | 0 |  | 666665.7 | 856161 |  | 840691.8 | 1211741 |
| 216690.2 | 263600.9 |  | 253180.2 | 765724.3 |  | 1283385 |  |  | 1425486 | 305192.5 |
| 365047.8 | 244943.6 |  | 286103.7 | 298538.2 |  | 907936 |  |  | 324597.5 | 374780 |
| 278138.5 | 524031.2 |  | 45760.81 | 575.1936 |  | 132246.6 |  |  | 778897.7 | 695354.1 |
| 175698.5 | 238778.4 |  | 662380.6 | 622448.1 |  | 996210.7 |  |  | 1596994 | 403267.2 |
| 243700.3 | 362265.2 |  |  | 71364.51 |  | 720987.3 |  |  | 864826.5 | 760389.6 |
| 271606 | 445364.6 |  |  | 731.3204 |  | 172473.5 |  |  | 649488 | 481387.6 |
| 227811.1 | 374928.9 |  |  | 32549.71 |  | 166839.9 |  |  | 427907.9 | 265481.7 |
| 203815.4 | 411817.9 |  |  | 5552.523 |  | 1076220 |  |  | 1046602 | 556895.9 |
| 136946.5 | 403770.1 |  |  | 0 |  | 1058572 |  |  | 1084853 | 377195.6 |
| 243679.9 | 274160.8 |  |  | 2231.751 |  | 483974.6 |  |  | 913991.3 | 464314.3 |
| 229113.2 | 290103.3 |  |  | 0 |  | 171001.1 |  |  | 470650.3 | 275531 |
| 181777.1 | 325407.9 |  |  | 288052.5 |  | 315590.1 |  |  | 288606.7 | 352380.3 |
| 308647.5 | 1592169 |  |  | 274513.8 |  | 728258.8 |  |  | 283549.4 | 240262.8 |
| 317918.8 | 608780.5 |  |  | 100666.4 |  | 666451.5 |  |  | 234840 | 363252.7 |
| 159776.7 | 497494.9 |  |  | 301548.9 |  | 616922.9 |  |  | 687599.7 | 216706.5 |
| 260749.3 | 367969.6 |  |  | 140453.2 |  | 1312402 |  |  | 135239.4 | 311607 |
| 171796.1 | 250902.6 |  |  | 540460.9 |  | 1256889 |  |  | 237983.3 | 147341.4 |
| 194430.5 | 402910.8 |  |  | 308614.2 |  | 214917 |  |  | 681730.6 | 277159.4 |
| 397490.5 | 446717.7 |  |  | 690.4299 |  | 903464.2 |  |  | 201542.8 | 740241.5 |
| 244337.8 | 574213.1 |  |  | 12628.23 |  | 1305715 |  |  | 241673.5 | 281049.2 |
| 220676 | 583701.1 |  |  | 0 |  | 879528.9 |  |  | 355241.9 | 431881.1 |
| 210084.3 | 1001047 |  |  | 322.123 |  | 782993.6 |  |  | 681590.5 | 645741 |
| 268962.2 | 255647.6 |  |  | 255.3698 |  | 969311.7 |  |  | 1512800 | 379877.7 |
| 363053.5 | 1106110 |  |  | 0 |  | 440801 |  |  | 413402.2 | 792361.4 |
| 191216.4 | 303240.9 |  |  | 404.3482 |  | 706704.2 |  |  | 367120.8 | 1460754 |
| 165622.2 | 455538.4 |  |  | 447677.3 |  | 1461579 |  |  | 238640.2 | 536541.1 |
| 208109.9 | 330446.7 |  |  | 36878.94 |  | 605760.4 |  |  | 470219.5 | 761615.6 |
| 178396.1 | 290326.9 |  |  | 16690.39 |  | 775666.4 |  |  | 336552.4 | 567878.3 |
| 280969 | 356731.4 |  |  |  |  |  |  |  | 543352.5 | 1322785 |
| 300277.2 | 367242.9 |  | L-Serine | |  | Stearic acid | |  | 482524.3 | 484309.5 |
| 174321.6 | 658673.7 |  | 443077.2 | 127355.8 |  | 71221.66 | 24243.22 |  | 494875.3 | 396469 |
| 221850.2 | 516930.9 |  | 159336.6 | 172898.8 |  | 129492.4 | 139863.3 |  | 186289.8 | 1035839 |
| 163783.2 | 425769.1 |  | 184777.1 | 202103.8 |  | 148699.3 | 128530.5 |  | 218532.5 | 335742.3 |
| 277675.8 | 338286.9 |  | 150526.9 | 290793.4 |  | 167700.3 | 80655.38 |  | 599158.8 | 546024.5 |
| 120726.7 | 607800.6 |  | 235384.9 | 336450 |  | 122719 | 164572.3 |  | 486680 | 962877.2 |
| 165427.1 | 525011.6 |  | 18485.27 | 483170.5 |  | 90620.39 | 108654.6 |  | 1038040 | 258332.3 |
| 96202.22 | 204416.3 |  | 652772.8 | 131098.7 |  | 74889.9 | 98896.43 |  | 856161 | 272896.5 |
| 261142.1 | 314979.1 |  | 900387.2 | 170075.5 |  | 91096.58 | 63521.94 |  |  | 670040.5 |
| 230640.6 | 327304.1 |  | 792970.8 | 86674.4 |  | 63271.7 | 66333.28 |  |  | 929962.5 |
| 341192.3 | 487237.1 |  | 140986.5 | 179014.3 |  | 106292.1 | 112182.6 |  |  | 1028106 |
| 116920.5 | 1042529 |  | 1657142 | 763728 |  | 91130.93 | 114037.4 |  |  | 820964.4 |
| 190178 |  |  | 435074.6 | 593167.8 |  | 81884.63 | 97393.65 |  |  | 1478826 |
| 166348.8 |  |  | 513070.8 | 481449.7 |  | 72807.41 | 63425.03 |  |  | 1047549 |
| 155480.5 |  |  | 903656.2 | 545178.8 |  | 89656.37 | 39440.27 |  |  | 303725 |
| 165797.2 |  |  | 265621.7 | 558878.6 |  | 52006.95 | 41698.63 |  |  | 481148.5 |
| 247289.8 |  |  | 171141.1 | 92931.71 |  | 78462.28 | 35327.81 |  |  | 311111.9 |
| 216343.2 |  |  | 218171 | 154565 |  | 24054.41 | 62979 |  |  | 367954.8 |
| 205346.6 |  |  | 163481.4 | 529443.3 |  | 78767.71 | 31443.55 |  |  | 378688.2 |
|  |  |  | 314555.8 | 137430.1 |  | 73474.32 | 33876.86 |  |  |  |
| L-Glutamine | |  | 263636.8 | 387337.8 |  | 19918.29 | 65401.84 |  | Stearic acid | |
| 500754.5 | 357652.9 |  | 213049.6 | 87496.96 |  | 57308.06 | 43476.13 |  | 24243.22 | 73005.49 |
| 10497 | 152678.8 |  | 500107.8 | 113690.7 |  | 59253.94 | 39065.71 |  | 139863.3 | 141360.2 |
| 13581.42 | 168004.3 |  | 119143.1 | 618672 |  | 42279.57 | 41172.96 |  | 128530.5 | 70363.21 |
| 87774.51 | 309899.9 |  | 221432.6 | 330259.2 |  | 162840.7 | 86251.34 |  | 80655.38 | 83987.53 |
| 109904.6 | 153400.4 |  | 261097.3 | 191978 |  | 88624.96 | 121073.6 |  | 164572.3 | 96431.21 |
| 3866.347 | 49513.41 |  | 371153.1 | 136466.2 |  | 71422.59 | 59364.16 |  | 108654.6 | 72298.62 |
| 19254.97 | 90505.13 |  | 68330.78 | 435292.1 |  | 85353.67 | 56833.36 |  | 98896.43 | 91344.18 |
| 23835.16 | 73736.5 |  | 338364.8 | 749.313 |  | 126264.2 | 25745.78 |  | 63521.94 | 72212.46 |
| 12047.01 | 157980.3 |  | 234060 | 255890.1 |  | 116384.6 | 69159.03 |  | 66333.28 | 62631.66 |
| 38588.42 | 179341.9 |  | 199614.6 | 277796.1 |  | 39628.96 | 62776.81 |  | 112182.6 | 66542.85 |
| 5575.717 | 731638.3 |  | 666512.4 | 142609.5 |  | 115000.6 | 74996.73 |  | 114037.4 | 35498.58 |
| 395609.3 | 256766.8 |  | 93683.41 | 286454.9 |  | 103337.1 | 57833.89 |  | 97393.65 | 63098.54 |
| 6692.703 | 352403.6 |  | 1972.345 | 194375.3 |  | 79853.9 | 67437 |  | 63425.03 | 42429.07 |
| 837.5199 | 577106.7 |  | 119001.1 | 82753.79 |  | 75414.99 | 24578.35 |  | 39440.27 | 53277.12 |
| 2139.436 | 236024.9 |  | 30092.07 | 164465.6 |  | 85506.19 | 33643.74 |  | 41698.63 | 44091.01 |
| 108674.8 | 372914.6 |  | 85962.86 | 466528.7 |  | 67127.31 | 77497.87 |  | 35327.81 | 57911.31 |
| 325901.7 | 536025 |  | 37084.03 | 240627.3 |  | 76754.29 | 92643.9 |  | 62979 | 43509.36 |
| 391723.1 | 207491.3 |  | 81228.55 | 282417.6 |  | 78265.52 | 145950.5 |  | 31443.55 | 48692.21 |
| 275992.8 | 351701.6 |  | 278516.9 | 2079.828 |  | 94621.88 | 107478.9 |  | 33876.86 | 29296.58 |
| 525326.8 | 315575.3 |  | 748996.4 | 1024309 |  | 134239.5 |  |  | 65401.84 | 45493.55 |
| 328805.8 | 64438.96 |  | 574267.4 | 244402.8 |  | 88397.02 |  |  | 43476.13 | 82980.05 |
| 636922.4 | 483572.1 |  | 234341 | 3177.728 |  | 45729.3 |  |  | 39065.71 | 48611.96 |
| 209163.5 | 886.3362 |  | 165090.8 | 260875.9 |  | 93243.21 |  |  | 41172.96 | 56195.14 |
| 1939.619 | 20734.63 |  |  | 92122.24 |  | 55972.32 |  |  | 86251.34 | 101907.6 |
| 65169.52 | 260518.8 |  |  | 2740.609 |  | 44798.9 |  |  | 121073.6 | 65938.19 |
| 73546.06 | 242200.2 |  |  | 4926.614 |  | 31046.96 |  |  | 59364.16 | 92055.81 |
| 200716.4 | 67568.96 |  |  | 32374.19 |  | 107711.5 |  |  | 56833.36 | 112928.2 |
| 65542.73 | 343277.7 |  |  | 2128.547 |  | 99933.02 |  |  | 25745.78 | 75886.91 |
| 198296.5 | 103061.8 |  |  | 18793.79 |  | 64680.8 |  |  | 69159.03 | 118490.8 |
| 168816 | 105835.8 |  |  | 4582.981 |  | 35603.43 |  |  | 62776.81 | 99927.21 |
| 65530.7 | 660145.1 |  |  | 219477.8 |  | 78675.14 |  |  | 74996.73 | 155143 |
| 159640.5 | 252270.8 |  |  | 213324.5 |  | 99002.77 |  |  | 57833.89 | 98828.11 |
| 169350.5 | 0 |  |  | 65597.03 |  | 73626.45 |  |  | 67437 | 91076.79 |
| 562769.1 | 240051.3 |  |  | 388865.4 |  | 94285.79 |  |  | 24578.35 | 113831.2 |
| 111151.6 | 849034.2 |  |  | 56011.71 |  | 123445.1 |  |  | 33643.74 | 81445.89 |
| 242117.2 | 212399.1 |  |  | 452914.1 |  | 109273.2 |  |  | 77497.87 | 112969.3 |
| 0 | 163252.4 |  |  | 130068.7 |  | 44346.67 |  |  | 92643.9 | 109404.8 |
| 0 | 256299.5 |  |  | 58.6182 |  | 70474.98 |  |  | 145950.5 | 53858.67 |
| 0 | 832017.6 |  |  | 6469.239 |  | 139613.9 |  |  | 107478.9 | 58306.46 |
| 0 | 733420.4 |  |  | 0 |  | 77403.57 |  |  |  | 84946.55 |
| 122878.1 | 748143 |  |  | 0 |  | 72726.68 |  |  |  | 109280.6 |
| 29945.12 | 431847.8 |  |  | 5264.188 |  | 93196.93 |  |  |  | 104373.2 |
| 0 | 380932.9 |  |  | 3195.348 |  | 52812.52 |  |  |  | 79762.77 |
| 108943.7 |  |  |  | 0 |  | 68562.23 |  |  |  | 153383.7 |
| 535383.5 |  |  |  | 637229.7 |  | 165159.5 |  |  |  | 93674.28 |
| 249032.6 |  |  |  | 88113.11 |  | 98452.07 |  |  |  | 50407.59 |
| 12158.05 |  |  |  | 141061 |  | 106940.6 |  |  |  | 78040.81 |
| 6686.043 |  |  |  |  |  |  |  |  |  | 47512.37 |
| 52916.97 |  |  | L-Glutamine | |  | Oleic acid | |  |  | 82888.84 |
| 52208.13 |  |  | 357652.9 | 60470 |  | 781777 | 47345.37 |  |  | 58658.39 |
|  |  |  | 152678.8 | 312847.5 |  | 638046.5 | 565744.6 |  |  |  |
| Creatine | |  | 168004.3 | 175810.6 |  | 404466 | 1278879 |  | Oleic acid | |
| 1065.411 | 78308.21 |  | 309899.9 | 69115.77 |  | 1209866 | 111984.4 |  | 47345.37 | 241167.3 |
| 47615.26 | 59143.11 |  | 153400.4 | 130183.8 |  | 597287.4 | 371801.6 |  | 565744.6 | 1138862 |
| 38160.2 | 2122.772 |  | 49513.41 | 126887.5 |  | 687674.4 | 899928.9 |  | 1278879 | 133400.8 |
| 57522.83 | 44864.37 |  | 90505.13 | 56752.57 |  | 209611.6 | 453435.2 |  | 111984.4 | 150645.7 |
| 61007.89 | 52060.62 |  | 73736.5 | 12518.56 |  | 323551.9 | 408270.5 |  | 371801.6 | 419767.7 |
| 45274.69 | 54275.78 |  | 157980.3 | 15143.93 |  | 161114.3 | 220009.2 |  | 899928.9 | 219206.3 |
| 37230.13 | 54256.95 |  | 179341.9 | 1351.894 |  | 981497 | 1160431 |  | 453435.2 | 444945.1 |
| 64378.85 | 45855.29 |  | 731638.3 | 60360.72 |  | 656982.6 | 975919.8 |  | 408270.5 | 219708 |
| 52190.22 | 92931.08 |  | 256766.8 | 64376.52 |  | 490789.9 | 1330469 |  | 220009.2 | 105278.4 |
| 62308 | 62456.9 |  | 352403.6 | 52815.29 |  | 404510.6 | 267560.4 |  | 1160431 | 344370.1 |
| 44389.45 | 122248.2 |  | 577106.7 | 80278.93 |  | 837116.4 | 180308.5 |  | 975919.8 | 228513.7 |
| 38809.48 | 128892.3 |  | 236024.9 | 170197.7 |  | 399599.4 | 236694.4 |  | 1330469 | 221847.5 |
| 55367.46 | 89506.19 |  | 372914.6 | 59200.55 |  | 561521.7 | 159705.5 |  | 267560.4 | 163914.9 |
| 43502.27 | 74174.65 |  | 536025 | 0 |  | 233679.6 | 543257.8 |  | 180308.5 | 222370.4 |
| 49027.91 | 65672.99 |  | 207491.3 | 219006 |  | 965945.9 | 47381.19 |  | 236694.4 | 85669.52 |
| 61096.38 | 56082.24 |  | 351701.6 | 123053.9 |  | 515116.1 | 116648.9 |  | 159705.5 | 123411.1 |
| 118547.2 | 110620.9 |  | 315575.3 | 102372.1 |  | 18757.05 | 503886.8 |  | 543257.8 | 57111.24 |
| 156044.4 | 116630.2 |  | 64438.96 | 141462.2 |  | 480278 | 99579.47 |  | 47381.19 | 131608.2 |
| 73777.06 | 84697.35 |  | 483572.1 | 143075.3 |  | 503592.6 | 135788 |  | 116648.9 | 33348.27 |
| 117102.4 | 50200.34 |  | 886.3362 | 246224.1 |  | 154883.6 | 227202.8 |  | 503886.8 | 83886.63 |
| 33196.52 | 1270.602 |  | 20734.63 | 128053.5 |  | 1731794 | 437915.7 |  | 99579.47 | 357068.3 |
| 55818.6 | 28723 |  | 260518.8 | 133579.6 |  | 663388.6 | 1136691 |  | 135788 | 91744.69 |
| 62771.33 | 215.7508 |  | 242200.2 | 146622.3 |  | 455021.8 | 300070.8 |  | 227202.8 | 201985 |
| 22536.83 | 4972.842 |  | 67568.96 | 179937.6 |  | 434684.4 | 130441.6 |  | 437915.7 | 383966.6 |
| 35240.6 | 18642.33 |  | 343277.7 | 0 |  | 700990.4 | 64139.67 |  | 1136691 | 138109.3 |
| 0 | 33805.82 |  | 103061.8 | 159580.7 |  | 662792.1 | 352114.6 |  | 300070.8 | 445123.9 |
| 26026.42 | 187.6082 |  | 105835.8 | 763946.1 |  | 41869.96 | 129474.2 |  | 130441.6 | 792584 |
| 737.4663 | 19438.2 |  | 660145.1 | 568751.6 |  | 614258.3 | 486854.4 |  | 64139.67 | 369621.8 |
| 27568.28 | 36486.82 |  | 252270.8 | 172401.4 |  | 896356.1 | 344501.5 |  | 352114.6 | 450673 |
| 16827.62 | 32118.4 |  | 0 | 46627.57 |  | 372629.2 | 291365.1 |  | 129474.2 | 248798.5 |
| 268.7651 | 72812.06 |  | 240051.3 | 158171.1 |  | 473371.2 | 88488.74 |  | 486854.4 | 960528.3 |
| 32499.26 | 1216.352 |  | 849034.2 | 59985.74 |  | 537439.3 | 68961.1 |  | 344501.5 | 278860 |
| 25125.39 | 1515.534 |  | 212399.1 | 277916.6 |  | 264877.9 | 282334.2 |  | 291365.1 | 132389 |
| 51186.82 | 749.0591 |  | 163252.4 | 113540.5 |  | 107587.9 | 330928.1 |  | 88488.74 | 642477.1 |
| 596.6619 | 977.823 |  | 256299.5 | 120092 |  | 221550.6 | 1003925 |  | 68961.1 | 108243.2 |
| 28364.79 | 591.0763 |  | 832017.6 | 7293.175 |  | 508955.2 | 894635.3 |  | 282334.2 | 215678.5 |
| 1517.066 | 15631.14 |  | 733420.4 | 551929 |  | 1126213 |  |  | 330928.1 | 512929.7 |
| 1007.199 | 1332.347 |  | 748143 | 200362.4 |  | 816322.8 |  |  | 1003925 | 78235.14 |
| 1485.078 | 30305.35 |  | 431847.8 | 689.7982 |  | 14513.66 |  |  | 894635.3 | 93392.77 |
| 1800.396 | 71711.44 |  | 380932.9 | 52882.04 |  | 875847.6 |  |  |  | 437887.3 |
| 786.6787 | 119229.7 |  |  | 104548.6 |  | 490465.8 |  |  |  | 553698.6 |
| 1616.437 | 68725.65 |  |  | 533.3888 |  | 52464.85 |  |  |  | 721866.1 |
| 2192.579 | 70180.42 |  |  | 39377.18 |  | 37998.37 |  |  |  | 322540.3 |
| 680.7938 |  |  |  | 10788.68 |  | 897096.6 |  |  |  | 949818 |
| 90155.85 |  |  |  | 7706.206 |  | 1012694 |  |  |  | 640931 |
| 600.5712 |  |  |  | 6142.344 |  | 314813.5 |  |  |  | 92107.01 |
| 70259.2 |  |  |  | 0 |  | 41892.86 |  |  |  | 246939.9 |
| 46162.58 |  |  |  | 264295.3 |  | 92110.88 |  |  |  | 126150.9 |
| 52093.44 |  |  |  | 54135.19 |  | 515216.7 |  |  |  | 144754.8 |
| 59664.32 |  |  |  | 108362.4 |  | 634039 |  |  |  | 182154.3 |
|  |  |  |  | 192258.7 |  | 416966.3 |  |  |  |  |
| Glutaric acid | |  |  | 135997.1 |  | 892847 |  |  | Linoleic acid | |
| 13326.93 | 17435.76 |  |  | 277868 |  | 919776.6 |  |  | 28342.67 | 158807.4 |
| 1875.249 | 3100.553 |  |  | 174588.9 |  | 57500.33 |  |  | 287256.9 | 514621.6 |
| 575.9397 | 23572.34 |  |  | 0 |  | 732526.2 |  |  | 396148.5 | 97824.14 |
| 2396.788 | 16284.76 |  |  | 46319.69 |  | 1160093 |  |  | 65748.8 | 110082.4 |
| 1269.029 | 15840.71 |  |  | 0 |  | 738206.9 |  |  | 232041.1 | 255168 |
| 151.3784 | 789.0209 |  |  | 0 |  | 493298 |  |  | 423991.2 | 131549.2 |
| 780.6394 | 29515.48 |  |  | 0 |  | 788430 |  |  | 309468.3 | 259346.8 |
| 1891.987 | 36961.83 |  |  | 0 |  | 318722.8 |  |  | 259150.2 | 131998.6 |
| 793.869 | 3582.551 |  |  | 0 |  | 910488.2 |  |  | 154076.2 | 75118.7 |
| 1664.979 | 4952.717 |  |  | 379087.2 |  | 1071848 |  |  | 485970.9 | 157652 |
| 199.4834 | 42158.32 |  |  | 183310.1 |  | 286818.6 |  |  | 351180.5 | 117263.2 |
| 2645.459 | 3917.39 |  |  | 116812.3 |  | 513829.6 |  |  | 308839.3 | 116681.6 |
| 2269.308 | 35631.48 |  |  |  |  |  |  |  | 98477.27 | 99421.52 |
| 0 | 44638.12 |  | Creatine | |  | Linoleic acid | |  | 83895.19 | 126745.2 |
| 1340.156 | 165712.1 |  | 78308.21 | 38251.82 |  | 469442.9 | 28342.67 |  | 69303.4 | 64697.1 |
| 5340.907 | 72549.51 |  | 59143.11 | 40752.1 |  | 380405.2 | 287256.9 |  | 89577.75 | 90516.38 |
| 3691.186 | 52809.6 |  | 2122.772 | 50969.83 |  | 290799.2 | 396148.5 |  | 337416.2 | 48283.55 |
| 3865.476 | 16492.7 |  | 44864.37 | 38792.4 |  | 806741.6 | 65748.8 |  | 40186.91 | 102743 |
| 546.9141 | 31962.78 |  | 52060.62 | 54561.31 |  | 407500.5 | 232041.1 |  | 64569.75 | 26761.97 |
| 2147.974 | 39931.45 |  | 54275.78 | 48753.36 |  | 299739.2 | 423991.2 |  | 348111.3 | 50855.04 |
| 14229.85 | 27926.58 |  | 54256.95 | 42928.23 |  | 138503.6 | 309468.3 |  | 98139.29 | 219544.3 |
| 1838.11 | 88296.27 |  | 45855.29 | 42349.26 |  | 186775.1 | 259150.2 |  | 87480.74 | 83646.01 |
| 1908.288 | 26956.79 |  | 92931.08 | 34157.43 |  | 88872.57 | 154076.2 |  | 115003.8 | 78143.66 |
| 526.967 | 4845.315 |  | 62456.9 | 37248.97 |  | 579497.2 | 485970.9 |  | 239424.7 | 158011.8 |
| 2536.38 | 19841.92 |  | 122248.2 | 459.0349 |  | 256237.2 | 351180.5 |  | 527740.6 | 86503.14 |
| 3924.062 | 10688.95 |  | 128892.3 | 94610 |  | 213151.9 | 308839.3 |  | 143742.7 | 198623.4 |
| 3667.182 | 29359.08 |  | 89506.19 | 59255.97 |  | 224408.1 | 98477.27 |  | 69100.75 | 276224.9 |
| 1634.692 | 35135.03 |  | 74174.65 | 51940.15 |  | 353675.6 | 83895.19 |  | 43707.96 | 162371.4 |
| 6265.01 | 10412.09 |  | 65672.99 | 53960.16 |  | 173921.8 | 69303.4 |  | 126324.4 | 168211 |
| 3782.621 | 16955.39 |  | 56082.24 | 70249.11 |  | 234476.2 | 89577.75 |  | 84290.63 | 136074.8 |
| 4829.622 | 16696.86 |  | 110620.9 | 39321.65 |  | 133981.4 | 337416.2 |  | 178328.9 | 449743.4 |
| 4938.184 | 37245.47 |  | 116630.2 | 72042.87 |  | 300967.5 | 40186.91 |  | 198754.5 | 146451.7 |
| 4325.898 | 24435.27 |  | 84697.35 | 91157.51 |  | 304730.7 | 64569.75 |  | 229521.4 | 101640.6 |
| 23438.36 | 197123.4 |  | 50200.34 | 49601.51 |  | 21747.45 | 348111.3 |  | 54890.15 | 253602.4 |
| 1435.315 | 122708.9 |  | 1270.602 | 58378.66 |  | 235831.3 | 98139.29 |  | 45923.96 | 74298.78 |
| 13047.83 | 211004.8 |  | 28723 | 53347.75 |  | 305287.1 | 87480.74 |  | 236085.6 | 171431 |
| 1970.777 | 521432 |  | 215.7508 | 63008.11 |  | 93562.12 | 115003.8 |  | 149893.5 | 205541.9 |
| 6306.85 | 53030.29 |  | 4972.842 | 24992.7 |  | 694143.7 | 239424.7 |  | 399925 | 54427.03 |
| 1676.29 | 163555.1 |  | 18642.33 | 18237.94 |  | 448838.3 | 527740.6 |  | 302391.6 | 60897.16 |
| 6035.063 | 20575.01 |  | 33805.82 | 1335.228 |  | 217437.7 | 143742.7 |  |  | 237745.7 |
| 12696.01 | 16239.97 |  | 187.6082 | 881.1732 |  | 283028.1 | 69100.75 |  |  | 262471.5 |
| 4706.316 | 29646.26 |  | 19438.2 | 396.36 |  | 442271.8 | 43707.96 |  |  | 306433.9 |
| 7607.679 | 35362.76 |  | 36486.82 | 1660.68 |  | 381966.9 | 126324.4 |  |  | 108227.3 |
| 16812.87 |  |  | 32118.4 | 282.5176 |  | 55956.02 | 84290.63 |  |  | 355137.7 |
| 3168.334 |  |  | 72812.06 | 1096.383 |  | 442962.8 | 178328.9 |  |  | 262849.2 |
| 14008.32 |  |  | 1216.352 | 26553.18 |  | 546498.7 | 198754.5 |  |  | 63995.41 |
| 2457.535 |  |  | 1515.534 | 1086.68 |  | 232533.2 | 229521.4 |  |  | 131137.5 |
| 341.0783 |  |  | 749.0591 | 65433.68 |  | 296695.1 | 54890.15 |  |  | 71230.59 |
| 2534.064 |  |  | 977.823 | 1737.188 |  | 255605.8 | 45923.96 |  |  | 106552.8 |
| 1997.827 |  |  | 591.0763 | 65616.31 |  | 145900.8 | 236085.6 |  |  | 101507.9 |
|  |  |  | 15631.14 | 1397.16 |  | 111575.5 | 149893.5 |  |  |  |
| Palmitic acid | |  | 1332.347 | 1257.263 |  | 197137.8 | 399925 |  | Cholesterol | |
| 539558.4 | 634080.9 |  | 30305.35 | 2108.547 |  | 219665.3 | 302391.6 |  | 394901.3 | 434355.3 |
| 1211741 | 388445.8 |  | 71711.44 | 22779.67 |  | 460283.5 |  |  | 842552 | 311087.1 |
| 305192.5 | 763563.4 |  | 119229.7 | 1160.946 |  | 355037.6 |  |  | 510397.5 | 184872.4 |
| 374780 | 806502.8 |  | 68725.65 | 563.73 |  | 13122.77 |  |  | 527399.2 | 384245 |
| 695354.1 | 955582.3 |  | 70180.42 | 705.8771 |  | 512431.7 |  |  | 931436.6 | 398510.5 |
| 403267.2 | 261469.6 |  |  | 54308.97 |  | 276310.9 |  |  | 507305 | 185397.4 |
| 760389.6 | 388101.6 |  |  | 1145.204 |  | 37951.8 |  |  | 382119.8 | 341462.6 |
| 481387.6 | 740338.8 |  |  | 1495.615 |  | 42081.09 |  |  | 242500.3 | 388611.5 |
| 265481.7 | 362625.1 |  |  | 1568.791 |  | 351913.6 |  |  | 366295.1 | 285320.7 |
| 556895.9 | 252847.4 |  |  | 1223.559 |  | 512355 |  |  | 318868.4 | 301910.3 |
| 377195.6 | 1533433 |  |  | 1057.803 |  | 174551.3 |  |  | 318968.4 | 318677.2 |
| 464314.3 | 796183.4 |  |  | 1097.899 |  | 31828.58 |  |  | 338940.7 | 329260.8 |
| 275531 | 588495.7 |  |  | 23645.6 |  | 65299.4 |  |  | 326996.5 | 246311.2 |
| 352380.3 | 745179.4 |  |  | 109861.6 |  | 157564.3 |  |  | 379062 | 240673.8 |
| 240262.8 | 3663175 |  |  | 455.6668 |  | 208355.8 |  |  | 552941.9 | 363531.2 |
| 363252.7 | 1127572 |  |  | 123900.7 |  | 184604.4 |  |  | 300155.5 | 419053 |
| 216706.5 | 1440474 |  |  | 62925.45 |  | 439466.1 |  |  | 398481.7 | 366673.9 |
| 311607 | 1311713 |  |  | 90924.27 |  | 441078.4 |  |  | 357184 | 293995.3 |
| 147341.4 | 597545.4 |  |  | 51900.99 |  | 51037.84 |  |  | 372503.2 | 187023.3 |
| 277159.4 | 1469992 |  |  | 3301.179 |  | 307410.9 |  |  | 336602 | 324310.4 |
| 740241.5 | 937603 |  |  | 992.7598 |  | 387360.5 |  |  | 427176.4 | 584419 |
| 281049.2 | 2032066 |  |  | 1061.69 |  | 339710.3 |  |  | 392452 | 247565.6 |
| 431881.1 | 663705.1 |  |  | 1168.181 |  | 219172.4 |  |  | 411138.4 | 345149 |
| 645741 | 631857.1 |  |  | 1683.19 |  | 277108.2 |  |  | 563135.2 | 278794.9 |
| 379877.7 | 1424121 |  |  | 2388.032 |  | 149849.7 |  |  | 314396.8 | 353458.5 |
| 792361.4 | 406570.2 |  |  | 2433.145 |  | 280318.5 |  |  | 438118.7 | 337732.4 |
| 1460754 | 628582.8 |  |  | 54345.53 |  | 553438.2 |  |  | 336519.9 | 306651.4 |
| 536541.1 | 715951.7 |  |  | 52460.34 |  | 177996.9 |  |  | 157367.8 | 341291.9 |
| 761615.6 | 623351.6 |  |  | 34075.38 |  | 310658.2 |  |  | 332368.3 | 228942 |
| 567878.3 | 1579819 |  |  |  |  |  |  |  | 400104.1 | 303032.4 |
| 1322785 | 741833.1 |  | Glutaric acid | |  | Urea | |  | 420791.5 | 225571.4 |
| 484309.5 | 901168.8 |  | 17435.76 | 4149.565 |  | 64867.14 | 97165.98 |  | 303168.6 | 332472.7 |
| 396469 | 568705.3 |  | 3100.553 | 2880.09 |  | 122836.2 | 268651.3 |  | 484732.1 | 178098 |
| 1035839 | 481847.9 |  | 23572.34 | 21970.99 |  | 132542.1 | 165088.1 |  | 475895 | 447758.3 |
| 335742.3 | 1018298 |  | 16284.76 | 4376.359 |  | 185916.6 | 194321.9 |  | 418838.5 | 225137.8 |
| 546024.5 | 1389642 |  | 15840.71 | 1596.298 |  | 155396.3 | 254763.4 |  | 486606.3 | 360610.3 |
| 962877.2 | 1827287 |  | 789.0209 | 950.9449 |  | 93425.76 | 111292.1 |  | 313743.7 | 384220.9 |
| 258332.3 | 848374.2 |  | 29515.48 | 3382.285 |  | 124857.3 | 200475.7 |  | 1006833 | 253512 |
| 272896.5 | 1069397 |  | 36961.83 | 1600.823 |  | 121999.7 | 59127.39 |  | 206471 | 345834.2 |
| 670040.5 | 404253.3 |  | 3582.551 | 2724.974 |  | 73304.24 | 199623.5 |  |  | 254742.2 |
| 929962.5 | 384181.3 |  | 4952.717 | 0 |  | 100976.2 | 21898.32 |  |  | 239300.6 |
| 1028106 | 351963.5 |  | 42158.32 | 0 |  | 34568.07 | 125547.8 |  |  | 105387 |
| 820964.4 | 1607255 |  | 3917.39 | 92.18038 |  | 44704.68 | 60780.78 |  |  | 266161.8 |
| 1478826 |  |  | 35631.48 | 2214.542 |  | 32301.18 | 40851.05 |  |  | 396687.8 |
| 1047549 |  |  | 44638.12 | 164.5386 |  | 34352.26 | 40483.09 |  |  | 359726.9 |
| 303725 |  |  | 165712.1 | 3926.071 |  | 45660.01 | 52109.5 |  |  | 258362.5 |
| 481148.5 |  |  | 72549.51 | 3648.909 |  | 79879.59 | 91712.76 |  |  | 382010.1 |
| 311111.9 |  |  | 52809.6 | 0 |  | 8365.662 | 47594.6 |  |  | 367943.8 |
| 367954.8 |  |  | 16492.7 | 2419.925 |  | 31092.85 | 46945.03 |  |  | 432619.6 |
| 378688.2 |  |  | 31962.78 | 2229.246 |  | 121190.3 | 40058.9 |  |  | 349744.7 |
|  |  |  | 39931.45 | 3568.154 |  | 158366.7 | 87903 |  |  |  |
| Oleic acid | |  | 27926.58 | 18240.27 |  | 180055.9 | 57559.99 |  | Malonic acid | |
| 241167.3 | 425403.1 |  | 88296.27 | 17807.84 |  | 211854.9 | 103701 |  | 2846897 | 2092946 |
| 1138862 | 244973.8 |  | 26956.79 | 407.9209 |  | 38172.06 | 40782.74 |  | 4330577 | 800561.7 |
| 133400.8 | 636328.5 |  | 4845.315 | 11873.25 |  | 172566.2 | 162572.5 |  | 2725879 | 2740984 |
| 150645.7 | 622172 |  | 19841.92 | 5577.254 |  | 246290.9 | 152605.1 |  | 4204368 | 3213241 |
| 419767.7 | 670117.3 |  | 10688.95 | 20691.83 |  | 202888.5 | 117609 |  | 8458857 | 3971297 |
| 219206.3 | 107738.1 |  | 29359.08 | 942.7511 |  | 347773.5 | 52652.35 |  | 2443088 | 2552371 |
| 444945.1 | 206003.5 |  | 35135.03 | 0 |  | 227500.4 | 38936.22 |  | 881481.3 | 3320934 |
| 219708 | 637752.1 |  | 10412.09 | 16640.17 |  | 196324.5 | 94263.11 |  | 2987025 | 3218196 |
| 105278.4 | 222972.4 |  | 16955.39 | 28751.33 |  | 273254.8 | 88874.19 |  | 1826324 | 1453320 |
| 344370.1 | 144870.7 |  | 16696.86 | 27214.65 |  | 149598.8 | 112745.7 |  | 2149543 | 1760669 |
| 228513.7 | 1329088 |  | 37245.47 | 13474.13 |  | 195956 | 21148.6 |  | 10929.48 | 976042 |
| 221847.5 | 661780.4 |  | 24435.27 | 5829.487 |  | 266168.1 | 309275.6 |  | 3202274 | 1269218 |
| 163914.9 | 429442.8 |  | 197123.4 | 2789.986 |  | 120242.6 | 36955.68 |  | 2091496 | 1069271 |
| 222370.4 | 483128.6 |  | 122708.9 | 16332.36 |  | 239133.5 | 35712.07 |  | 3286684 | 2342883 |
| 85669.52 | 3775507 |  | 211004.8 | 14811.1 |  | 167634.7 | 111886.7 |  | 3163269 | 363787.5 |
| 123411.1 | 965998.6 |  | 521432 | 14165.53 |  | 453644.1 | 134066.9 |  | 4376930 | 1876608 |
| 57111.24 | 886600.5 |  | 53030.29 | 6153.958 |  | 170497.9 | 214205.3 |  | 3469278 | 2514134 |
| 131608.2 | 1146965 |  | 163555.1 | 10820.15 |  | 243018.8 | 113349.5 |  | 3684625 | 2667219 |
| 33348.27 | 412062.9 |  | 20575.01 | 20457.81 |  | 355717.4 |  |  | 5162508 | 1316516 |
| 83886.63 | 1345366 |  | 16239.97 | 18661.06 |  | 387917 |  |  | 3424326 | 2936890 |
| 357068.3 | 686874.7 |  | 29646.26 | 3510.452 |  | 88550.91 |  |  | 3975650 | 1021960 |
| 91744.69 | 1863796 |  | 35362.76 | 5318.786 |  | 396337.1 |  |  | 5126086 | 1241487 |
| 201985 | 542177 |  |  | 16623.49 |  | 268559.5 |  |  | 3528628 | 1716899 |
| 383966.6 | 670688.8 |  |  | 4759.636 |  | 218727.3 |  |  | 17681.38 | 3528066 |
| 138109.3 | 968700.1 |  |  | 9603.51 |  | 182811.4 |  |  | 3088781 | 622781.7 |
| 445123.9 | 163006.7 |  |  | 13142.28 |  | 300083.9 |  |  | 5154530 | 216979.3 |
| 792584 | 387611.8 |  |  | 30651.19 |  | 224008.2 |  |  | 5091526 | 1360389 |
| 369621.8 | 441119.9 |  |  | 15596.21 |  | 295498.6 |  |  | 2872056 | 801360.4 |
| 450673 | 372114.5 |  |  | 551.416 |  | 254116.6 |  |  | 3837795 | 1310806 |
| 248798.5 | 1383111 |  |  | 27098.84 |  | 291864.6 |  |  | 2710017 | 1380413 |
| 960528.3 | 404032.4 |  |  | 10068.66 |  | 183752.5 |  |  | 5283062 | 983203.2 |
| 278860 | 568100.2 |  |  | 11898.09 |  | 256501.2 |  |  | 3964662 | 9782.07 |
| 132389 | 287804.7 |  |  | 2834.248 |  | 217590.2 |  |  | 106725.5 | 597619.3 |
| 642477.1 | 181509.7 |  |  | 15271.46 |  | 211868.1 |  |  | 3742771 | 1076192 |
| 108243.2 | 653157.9 |  |  | 34152.39 |  | 432643.7 |  |  | 4127092 | 604818.7 |
| 215678.5 | 672726.3 |  |  | 39476.22 |  | 263746.6 |  |  | 4924161 | 1338376 |
| 512929.7 | 1427640 |  |  | 14320.92 |  | 113201.7 |  |  | 3912131 | 105877.7 |
| 78235.14 | 578388.9 |  |  | 32564.32 |  | 262390.8 |  |  | 1790353 | 18086.47 |
| 93392.77 | 723295.1 |  |  | 778.6503 |  | 417.6717 |  |  | 4656410 | 100754.8 |
| 437887.3 | 214418.3 |  |  | 3154.901 |  | 216534.5 |  |  |  | 15233.85 |
| 553698.6 | 189551.4 |  |  | 12546.35 |  | 372842 |  |  |  | 592808.5 |
| 721866.1 | 172802.7 |  |  | 786.2087 |  | 435275.8 |  |  |  | 19672.15 |
| 322540.3 | 1376278 |  |  | 1425.26 |  | 146735.9 |  |  |  | 14375.12 |
| 949818 |  |  |  | 38556.06 |  | 528576.7 |  |  |  | 429899.1 |
| 640931 |  |  |  | 6645.669 |  | 128001.3 |  |  |  | 2281888 |
| 92107.01 |  |  |  | 1151.365 |  | 162664.7 |  |  |  | 1057504 |
| 246939.9 |  |  |  |  |  |  |  |  |  | 2933523 |
| 126150.9 |  |  | Palmitic acid | |  | Malonic acid | |  |  | 1080897 |
| 144754.8 |  |  | 634080.9 | 954176.6 |  | 457812.7 | 2846897 |  |  | 3680682 |
| 182154.3 |  |  | 388445.8 | 1058206 |  | 2100123 | 4330577 |  |  | 2659544 |
|  |  |  | 763563.4 | 784939.5 |  | 3148951 | 2725879 |  |  |  |
| Linoleic acid | |  | 806502.8 | 1841482 |  | 4023218 | 4204368 |  |  |  |
| 158807.4 | 153125.1 |  | 955582.3 | 963980.6 |  | 3878230 | 8458857 |  |  |  |
| 514621.6 | 93281.39 |  | 261469.6 | 878557.9 |  | 3445549 | 2443088 |  |  |  |
| 97824.14 | 218964.4 |  | 388101.6 | 384235.7 |  | 3201486 | 881481.3 |  |  |  |
| 110082.4 | 205155.5 |  | 740338.8 | 605243.5 |  | 2916050 | 2987025 |  |  |  |
| 255168 | 208178.8 |  | 362625.1 | 341774 |  | 3190247 | 1826324 |  |  |  |
| 131549.2 | 48687.66 |  | 252847.4 | 1212022 |  | 1400904 | 2149543 |  |  |  |
| 259346.8 | 73853.88 |  | 1533433 | 825181.3 |  | 2081974 | 10929.48 |  |  |  |
| 131998.6 | 192993.1 |  | 796183.4 | 549900.4 |  | 1970048 | 3202274 |  |  |  |
| 75118.7 | 104063.2 |  | 588495.7 | 644968 |  | 2005945 | 2091496 |  |  |  |
| 157652 | 84684.15 |  | 745179.4 | 971845.8 |  | 2498272 | 3286684 |  |  |  |
| 117263.2 | 583912.4 |  | 3663175 | 540449 |  | 1905306 | 3163269 |  |  |  |
| 116681.6 | 273991 |  | 1127572 | 708358.7 |  | 2460360 | 4376930 |  |  |  |
| 99421.52 | 157574.4 |  | 1440474 | 338938 |  | 2172809 | 3469278 |  |  |  |
| 126745.2 | 146281.1 |  | 1311713 | 1206103 |  | 2460725 | 3684625 |  |  |  |
| 64697.1 | 1138746 |  | 597545.4 | 762911.5 |  | 2485567 | 5162508 |  |  |  |
| 90516.38 | 405614 |  | 1469992 | 103386.3 |  | 2566984 | 3424326 |  |  |  |
| 48283.55 | 338340.5 |  | 937603 | 691448.3 |  | 572052.6 | 3975650 |  |  |  |
| 102743 | 324012.3 |  | 2032066 | 743386.2 |  | 1138335 | 5126086 |  |  |  |
| 26761.97 | 157446 |  | 663705.1 | 293279.9 |  | 3317039 | 3528628 |  |  |  |
| 50855.04 | 459667.1 |  | 631857.1 | 1891353 |  | 1219953 | 17681.38 |  |  |  |
| 219544.3 | 235325.6 |  | 1424121 | 961090.8 |  | 1766270 | 3088781 |  |  |  |
| 83646.01 | 676573.6 |  | 406570.2 | 595423 |  | 1898992 | 5154530 |  |  |  |
| 78143.66 | 215203.3 |  | 628582.8 | 585423.6 |  | 1960061 | 5091526 |  |  |  |
| 158011.8 | 193360.3 |  | 715951.7 | 953696.2 |  | 673552.1 | 2872056 |  |  |  |
| 86503.14 | 357740.3 |  | 623351.6 | 1002574 |  | 1266352 | 3837795 |  |  |  |
| 198623.4 | 72645.9 |  | 1579819 | 174822.1 |  | 1762392 | 2710017 |  |  |  |
| 276224.9 | 134767 |  | 741833.1 | 953046.3 |  | 2268053 | 5283062 |  |  |  |
| 162371.4 | 195102.2 |  | 901168.8 | 1222195 |  | 1974600 | 3964662 |  |  |  |
| 168211 | 159813.9 |  | 568705.3 | 655415.3 |  | 911672.6 | 106725.5 |  |  |  |
| 136074.8 | 431576.5 |  | 481847.9 | 742424.7 |  | 1283766 | 3742771 |  |  |  |
| 449743.4 | 146649.9 |  | 1018298 | 850168.6 |  | 718938.2 | 4127092 |  |  |  |
| 146451.7 | 250588.4 |  | 1389642 | 434195.3 |  | 1157661 | 4924161 |  |  |  |
| 101640.6 | 119900.3 |  | 1827287 | 336065.1 |  | 614812.5 | 3912131 |  |  |  |
| 253602.4 | 135046.4 |  | 848374.2 | 385518.7 |  | 1962231 | 1790353 |  |  |  |
| 74298.78 | 250886.8 |  | 1069397 | 666665.7 |  | 32607.94 | 4656410 |  |  |  |
| 171431 | 356356.3 |  | 404253.3 | 1283385 |  | 2941515 |  |  |  |  |
| 205541.9 | 490341.6 |  | 384181.3 | 907936 |  | 541081.8 |  |  |  |  |
| 54427.03 | 169038 |  | 351963.5 | 132246.6 |  | 534207.6 |  |  |  |  |
| 60897.16 | 245790.8 |  | 1607255 | 996210.7 |  | 1039366 |  |  |  |  |
| 237745.7 | 81164.91 |  |  | 720987.3 |  | 23160.51 |  |  |  |  |
| 262471.5 | 90186.28 |  |  | 172473.5 |  | 572975.9 |  |  |  |  |
| 306433.9 | 73872.89 |  |  | 166839.9 |  | 24405.91 |  |  |  |  |
| 108227.3 | 343120.9 |  |  | 1076220 |  | 451072.3 |  |  |  |  |
| 355137.7 |  |  |  | 1058572 |  | 246485.4 |  |  |  |  |
| 262849.2 |  |  |  | 483974.6 |  | 383322 |  |  |  |  |
| 63995.41 |  |  |  | 171001.1 |  | 362113.9 |  |  |  |  |
| 131137.5 |  |  |  | 315590.1 |  | 1748309 |  |  |  |  |
| 71230.59 |  |  |  | 728258.8 |  | 2360797 |  |  |  |  |
| 106552.8 |  |  |  | 666451.5 |  | 838029 |  |  |  |  |
| 101507.9 |  |  |  | 616922.9 |  | 3389355 |  |  |  |  |
|  |  |  |  | 1312402 |  | 434903.5 |  |  |  |  |
| Cholesterol | |  |  | 1256889 |  | 1503222 |  |  |  |  |
| 434355.3 | 480924.1 |  |  | 214917 |  | 1817524 |  |  |  |  |
| 311087.1 | 311737.7 |  |  | 903464.2 |  | 61994.03 |  |  |  |  |
| 184872.4 | 270239.8 |  |  | 1305715 |  | 710321.2 |  |  |  |  |
| 384245 | 309801.4 |  |  | 879528.9 |  | 24983.29 |  |  |  |  |
| 398510.5 | 379395.3 |  |  | 782993.6 |  | 491407.9 |  |  |  |  |
| 185397.4 | 233849.1 |  |  | 969311.7 |  | 539075.5 |  |  |  |  |
| 341462.6 | 330521.9 |  |  | 440801 |  | 97039.04 |  |  |  |  |
| 388611.5 | 314814.9 |  |  | 706704.2 |  | 291664.7 |  |  |  |  |
| 285320.7 | 466312.7 |  |  | 1461579 |  | 4442772 |  |  |  |  |
| 301910.3 | 373443.7 |  |  | 605760.4 |  | 3341357 |  |  |  |  |
| 318677.2 | 443230.5 |  |  | 775666.4 |  | 2946584 |  |  |  |  |
| 329260.8 | 612659.6 |  |  |  |  |  |  |  |  |  |
| 246311.2 | 302319.8 |  | Stearic acid | |  |  |  |  |  |  |
| 240673.8 | 322593.3 |  | 82956.38 | 71221.66 |  |  |  |  |  |  |
| 363531.2 | 762043.1 |  | 66342.98 | 129492.4 |  |  |  |  |  |  |
| 419053 | 436651.2 |  | 112075.2 | 148699.3 |  |  |  |  |  |  |
| 366673.9 | 516259 |  | 92360.59 | 167700.3 |  |  |  |  |  |  |
| 293995.3 | 416559 |  | 148606.5 | 122719 |  |  |  |  |  |  |
| 187023.3 | 331252 |  | 53308.25 | 90620.39 |  |  |  |  |  |  |
| 324310.4 | 433517 |  | 46922.51 | 74889.9 |  |  |  |  |  |  |
| 584419 | 307216 |  | 59919.92 | 91096.58 |  |  |  |  |  |  |
| 247565.6 | 756037.7 |  | 47565.59 | 63271.7 |  |  |  |  |  |  |
| 345149 | 329264.1 |  | 39798.02 | 106292.1 |  |  |  |  |  |  |
| 278794.9 | 304797.7 |  | 131748.1 | 91130.93 |  |  |  |  |  |  |
| 353458.5 | 346601.7 |  | 77921.55 | 81884.63 |  |  |  |  |  |  |
| 337732.4 | 296711.2 |  | 67188.45 | 72807.41 |  |  |  |  |  |  |
| 306651.4 | 193663 |  | 74591.85 | 89656.37 |  |  |  |  |  |  |
| 341291.9 | 400398.9 |  | 168556.3 | 52006.95 |  |  |  |  |  |  |
| 228942 | 357284.5 |  | 105792.8 | 78462.28 |  |  |  |  |  |  |
| 303032.4 | 374985.4 |  | 124413.6 | 24054.41 |  |  |  |  |  |  |
| 225571.4 | 398673.5 |  | 130024.4 | 78767.71 |  |  |  |  |  |  |
| 332472.7 | 330864.6 |  | 64159.97 | 73474.32 |  |  |  |  |  |  |
| 178098 | 200048.3 |  | 131637.3 | 19918.29 |  |  |  |  |  |  |
| 447758.3 | 153783.2 |  | 98381.8 | 57308.06 |  |  |  |  |  |  |
| 225137.8 | 447271.3 |  | 217664.7 | 59253.94 |  |  |  |  |  |  |
| 360610.3 | 425931.2 |  | 79942 | 42279.57 |  |  |  |  |  |  |
| 384220.9 | 263849.7 |  | 68689.43 | 162840.7 |  |  |  |  |  |  |
| 253512 | 376714.6 |  | 142496.7 | 88624.96 |  |  |  |  |  |  |
| 345834.2 | 330710.4 |  | 57351.09 | 71422.59 |  |  |  |  |  |  |
| 254742.2 | 243348.7 |  | 86434.14 | 85353.67 |  |  |  |  |  |  |
| 239300.6 | 421686.9 |  | 106206.5 | 126264.2 |  |  |  |  |  |  |
| 105387 | 431386.4 |  | 82375.79 | 116384.6 |  |  |  |  |  |  |
| 266161.8 | 435778.4 |  | 169936.2 | 39628.96 |  |  |  |  |  |  |
| 396687.8 |  |  | 93990.18 | 115000.6 |  |  |  |  |  |  |
| 359726.9 |  |  | 128044.4 | 103337.1 |  |  |  |  |  |  |
| 258362.5 |  |  | 77826.97 | 79853.9 |  |  |  |  |  |  |
| 382010.1 |  |  | 67616.23 | 75414.99 |  |  |  |  |  |  |
| 367943.8 |  |  | 116555.5 | 85506.19 |  |  |  |  |  |  |
| 432619.6 |  |  | 139233.8 | 67127.31 |  |  |  |  |  |  |
| 349744.7 |  |  | 194512.7 | 76754.29 |  |  |  |  |  |  |
|  |  |  | 113544.3 | 78265.52 |  |  |  |  |  |  |
| Urea | |  | 108233.3 | 94621.88 |  |  |  |  |  |  |
| 111063.4 | 90691.06 |  | 49738.51 | 134239.5 |  |  |  |  |  |  |
| 188251.9 | 154262.4 |  | 55036.85 | 88397.02 |  |  |  |  |  |  |
| 93368.45 | 176425.5 |  | 65339.54 | 45729.3 |  |  |  |  |  |  |
| 45501.51 | 127231.8 |  | 119561.9 | 93243.21 |  |  |  |  |  |  |
| 32308.4 | 152938.9 |  |  | 55972.32 |  |  |  |  |  |  |
| 87865.79 | 87294.41 |  |  | 44798.9 |  |  |  |  |  |  |
| 129412.8 | 18295.89 |  |  | 31046.96 |  |  |  |  |  |  |
| 145096.3 | 12148.22 |  |  | 107711.5 |  |  |  |  |  |  |
| 116662.7 | 58609.2 |  |  | 99933.02 |  |  |  |  |  |  |
| 5362.979 | 113976.1 |  |  | 64680.8 |  |  |  |  |  |  |
| 26146.06 | 41719.83 |  |  | 35603.43 |  |  |  |  |  |  |
| 54920.71 | 119252.6 |  |  | 78675.14 |  |  |  |  |  |  |
| 44657.03 | 83622.64 |  |  | 99002.77 |  |  |  |  |  |  |
| 33614.42 | 46087.22 |  |  | 73626.45 |  |  |  |  |  |  |
| 122610.8 | 349427 |  |  | 94285.79 |  |  |  |  |  |  |
| 166024 | 167680.9 |  |  | 123445.1 |  |  |  |  |  |  |
| 94353.82 | 169417.3 |  |  | 109273.2 |  |  |  |  |  |  |
| 133310.8 | 207875.7 |  |  | 44346.67 |  |  |  |  |  |  |
| 102105.9 | 118441.1 |  |  | 70474.98 |  |  |  |  |  |  |
| 48812.63 | 115944.9 |  |  | 139613.9 |  |  |  |  |  |  |
| 232548.3 | 192444.2 |  |  | 77403.57 |  |  |  |  |  |  |
| 129676.4 | 309050.8 |  |  | 72726.68 |  |  |  |  |  |  |
| 37047.55 | 153837.8 |  |  | 93196.93 |  |  |  |  |  |  |
| 142573.3 | 218925.2 |  |  | 52812.52 |  |  |  |  |  |  |
| 108954.6 | 133561.6 |  |  | 68562.23 |  |  |  |  |  |  |
| 234778.4 | 257969.9 |  |  | 165159.5 |  |  |  |  |  |  |
| 160354.5 | 119447.7 |  |  | 98452.07 |  |  |  |  |  |  |
| 132700.1 | 231906.2 |  |  | 106940.6 |  |  |  |  |  |  |
| 178306 | 173967.4 |  |  |  |  |  |  |  |  |  |
| 186639 | 217655.7 |  | Cholesterol | |  |  |  |  |  |  |
| 155546.5 | 164680.7 |  | 480924.1 | 269253.1 |  |  |  |  |  |  |
| 160165.1 | 247497.2 |  | 311737.7 | 247301.2 |  |  |  |  |  |  |
| 152643.7 | 229142.7 |  | 270239.8 | 336904.4 |  |  |  |  |  |  |
| 127692.2 | 212000.5 |  | 309801.4 | 571462.8 |  |  |  |  |  |  |
| 121708 | 36266.8 |  | 379395.3 | 521813.9 |  |  |  |  |  |  |
| 202985.4 | 226559.9 |  | 233849.1 | 301312.2 |  |  |  |  |  |  |
| 202336.4 | 278228.6 |  | 330521.9 | 209063.5 |  |  |  |  |  |  |
| 19880.85 | 297037 |  | 314814.9 | 332824.7 |  |  |  |  |  |  |
| 153887.8 | 259632.7 |  | 466312.7 | 303819.6 |  |  |  |  |  |  |
| 22132.92 | 27527.56 |  | 373443.7 | 390902.3 |  |  |  |  |  |  |
| 211484.5 | 51783.03 |  | 443230.5 | 202841.1 |  |  |  |  |  |  |
| 157578.2 | 209733 |  | 612659.6 | 142023 |  |  |  |  |  |  |
| 197693 | 242009.2 |  | 302319.8 | 347568.7 |  |  |  |  |  |  |
| 161396.1 |  |  | 322593.3 | 787984.1 |  |  |  |  |  |  |
| 39447.81 |  |  | 762043.1 | 298021.1 |  |  |  |  |  |  |
| 184916.3 |  |  | 436651.2 | 418379.4 |  |  |  |  |  |  |
| 21884.45 |  |  | 516259 | 74474.91 |  |  |  |  |  |  |
| 89040.86 |  |  | 416559 | 418005.7 |  |  |  |  |  |  |
| 46669.16 |  |  | 331252 | 383721 |  |  |  |  |  |  |
| 64578.42 |  |  | 433517 | 314911.2 |  |  |  |  |  |  |
|  |  |  | 307216 | 304226.3 |  |  |  |  |  |  |
| Phosphoric acid | |  | 756037.7 | 407653.1 |  |  |  |  |  |  |
| 63810.3 | 39908.2 |  | 329264.1 | 169000.6 |  |  |  |  |  |  |
| 9851.688 | 34505.55 |  | 304797.7 | 588071.7 |  |  |  |  |  |  |
| 7964.79 | 12192.26 |  | 346601.7 | 368080.8 |  |  |  |  |  |  |
| 9672.9 | 29901.24 |  | 296711.2 | 252296.4 |  |  |  |  |  |  |
| 3739.78 | 36224.02 |  | 193663 | 481190.6 |  |  |  |  |  |  |
| 3121.422 | 2643.222 |  | 400398.9 | 327492.5 |  |  |  |  |  |  |
| 9046.908 | 27338.53 |  | 357284.5 | 282638.3 |  |  |  |  |  |  |
| 6078.668 | 20336.83 |  | 374985.4 | 382554.4 |  |  |  |  |  |  |
| 7430 | 52458.89 |  | 398673.5 | 387155.6 |  |  |  |  |  |  |
| 2965.736 | 21663.98 |  | 330864.6 | 400448.5 |  |  |  |  |  |  |
| 1870.21 | 42147.62 |  | 200048.3 | 387372.2 |  |  |  |  |  |  |
| 35680.67 | 11820.86 |  | 153783.2 | 400649.1 |  |  |  |  |  |  |
| 3196.512 | 31524.96 |  | 447271.3 | 227593 |  |  |  |  |  |  |
| 6022.018 | 62642.84 |  | 425931.2 | 414368.2 |  |  |  |  |  |  |
| 7675.131 | 50572.13 |  | 263849.7 | 311244 |  |  |  |  |  |  |
| 43797.61 | 30580.4 |  | 376714.6 | 438678.3 |  |  |  |  |  |  |
| 31011.35 | 78493.47 |  | 330710.4 | 430760.9 |  |  |  |  |  |  |
| 30538.24 | 36245.36 |  | 243348.7 | 455198.4 |  |  |  |  |  |  |
| 30577.44 | 2253.246 |  | 421686.9 | 368110.5 |  |  |  |  |  |  |
| 36155.4 | 37307.77 |  | 431386.4 | 41031.36 |  |  |  |  |  |  |
| 82323.27 | 33070.39 |  | 435778.4 | 438553.6 |  |  |  |  |  |  |
| 99643.89 | 50461.52 |  |  | 418941.8 |  |  |  |  |  |  |
| 10703.34 | 3492.102 |  |  | 388314.8 |  |  |  |  |  |  |
| 1034.09 | 6290.713 |  |  | 427701.8 |  |  |  |  |  |  |
| 6199.121 | 30801.06 |  |  | 396164.1 |  |  |  |  |  |  |
| 11791.09 | 21989.62 |  |  | 442770 |  |  |  |  |  |  |
| 40789.91 | 24014.87 |  |  | 232576.7 |  |  |  |  |  |  |
| 27874.63 | 51503.96 |  |  | 146386.4 |  |  |  |  |  |  |
| 30836.71 | 33642.05 |  |  | 0 |  |  |  |  |  |  |
| 27403.79 | 36480.93 |  |  | 19962.96 |  |  |  |  |  |  |
| 23159.26 | 167931.2 |  |  | 46605.84 |  |  |  |  |  |  |
| 45465.63 | 76037.88 |  |  | 71144.36 |  |  |  |  |  |  |
| 31910 | 75928.36 |  |  | 535856.6 |  |  |  |  |  |  |
| 46468.45 | 171549 |  |  | 52623.9 |  |  |  |  |  |  |
| 19225.87 | 59911.15 |  |  | 0 |  |  |  |  |  |  |
| 54569.17 | 128446.1 |  |  | 466476.6 |  |  |  |  |  |  |
| 21699.71 | 95773.85 |  |  | 24310.16 |  |  |  |  |  |  |
| 16135.73 | 78120.81 |  |  | 379852.9 |  |  |  |  |  |  |
| 4807.937 | 74603.11 |  |  | 40098.79 |  |  |  |  |  |  |
| 9669.872 | 33793.56 |  |  | 442906.5 |  |  |  |  |  |  |
| 22772.69 | 44504.66 |  |  | 272125 |  |  |  |  |  |  |
| 14331.81 | 30295.54 |  |  | 284889.5 |  |  |  |  |  |  |
| 5147.807 | 25204.07 |  |  | 873752.5 |  |  |  |  |  |  |
| 29180.94 |  |  |  | 395420.9 |  |  |  |  |  |  |
| 30237.72 |  |  |  | 479428.5 |  |  |  |  |  |  |
| 23217.54 |  |  |  |  |  |  |  |  |  |  |
| 4319.44 |  |  | Phosphoric acid | |  |  |  |  |  |  |
| 812.7602 |  |  | 39908.2 | 4678.824 |  |  |  |  |  |  |
| 8021.421 |  |  | 34505.55 | 34443.58 |  |  |  |  |  |  |
| 10533.95 |  |  | 12192.26 | 34163.36 |  |  |  |  |  |  |
|  |  |  | 29901.24 | 38634.06 |  |  |  |  |  |  |
|  |  |  | 36224.02 | 35816.53 |  |  |  |  |  |  |
|  |  |  | 2643.222 | 16508.6 |  |  |  |  |  |  |
|  |  |  | 27338.53 | 11293.04 |  |  |  |  |  |  |
|  |  |  | 20336.83 | 5859.356 |  |  |  |  |  |  |
|  |  |  | 52458.89 | 6083.421 |  |  |  |  |  |  |
|  |  |  | 21663.98 | 6061.459 |  |  |  |  |  |  |
|  |  |  | 42147.62 | 0 |  |  |  |  |  |  |
|  |  |  | 11820.86 | 0 |  |  |  |  |  |  |
|  |  |  | 31524.96 | 24427.61 |  |  |  |  |  |  |
|  |  |  | 62642.84 | 20789.08 |  |  |  |  |  |  |
|  |  |  | 50572.13 | 22931.06 |  |  |  |  |  |  |
|  |  |  | 30580.4 | 6166.133 |  |  |  |  |  |  |
|  |  |  | 78493.47 | 0 |  |  |  |  |  |  |
|  |  |  | 36245.36 | 2927.826 |  |  |  |  |  |  |
|  |  |  | 2253.246 | 5290.843 |  |  |  |  |  |  |
|  |  |  | 37307.77 | 6979.815 |  |  |  |  |  |  |
|  |  |  | 33070.39 | 6001.054 |  |  |  |  |  |  |
|  |  |  | 50461.52 | 356.5912 |  |  |  |  |  |  |
|  |  |  | 3492.102 | 13059.26 |  |  |  |  |  |  |
|  |  |  | 6290.713 | 31224.24 |  |  |  |  |  |  |
|  |  |  | 30801.06 | 11478.69 |  |  |  |  |  |  |
|  |  |  | 21989.62 | 9635.909 |  |  |  |  |  |  |
|  |  |  | 24014.87 | 1506.109 |  |  |  |  |  |  |
|  |  |  | 51503.96 | 0 |  |  |  |  |  |  |
|  |  |  | 33642.05 | 22523.63 |  |  |  |  |  |  |
|  |  |  | 36480.93 | 35804.49 |  |  |  |  |  |  |
|  |  |  | 167931.2 | 46960.16 |  |  |  |  |  |  |
|  |  |  | 76037.88 | 19386.19 |  |  |  |  |  |  |
|  |  |  | 75928.36 | 12264.6 |  |  |  |  |  |  |
|  |  |  | 171549 | 5018.65 |  |  |  |  |  |  |
|  |  |  | 59911.15 | 7702.818 |  |  |  |  |  |  |
|  |  |  | 128446.1 | 26953.52 |  |  |  |  |  |  |
|  |  |  | 95773.85 | 18135.1 |  |  |  |  |  |  |
|  |  |  | 78120.81 | 11385.59 |  |  |  |  |  |  |
|  |  |  | 74603.11 | 2915.019 |  |  |  |  |  |  |
|  |  |  | 33793.56 | 13999.39 |  |  |  |  |  |  |
|  |  |  | 44504.66 | 12045.18 |  |  |  |  |  |  |
|  |  |  | 30295.54 | 4541.11 |  |  |  |  |  |  |
|  |  |  | 25204.07 | 8851.405 |  |  |  |  |  |  |
|  |  |  |  | 8860.604 |  |  |  |  |  |  |
|  |  |  |  | 3270.096 |  |  |  |  |  |  |
|  |  |  |  | 10030.21 |  |  |  |  |  |  |
|  |  |  |  | 4193.286 |  |  |  |  |  |  |
|  |  |  |  | 4614.439 |  |  |  |  |  |  |
|  |  |  |  | 7655.423 |  |  |  |  |  |  |
|  |  |  |  | 778.9111 |  |  |  |  |  |  |
|  |  |  |  | 1192.207 |  |  |  |  |  |  |
|  |  |  |  | 27641.78 |  |  |  |  |  |  |
|  |  |  |  | 11285.68 |  |  |  |  |  |  |
|  |  |  |  | 31426.44 |  |  |  |  |  |  |
|  |  |  |  | 5527.014 |  |  |  |  |  |  |
|  |  |  |  | 22624.29 |  |  |  |  |  |  |
|  |  |  |  | 21660.52 |  |  |  |  |  |  |
|  |  |  |  | 1385.809 |  |  |  |  |  |  |
|  |  |  |  | 3580.527 |  |  |  |  |  |  |
|  |  |  |  | 917.1193 |  |  |  |  |  |  |
|  |  |  |  | 471.1534 |  |  |  |  |  |  |
|  |  |  |  | 1269.863 |  |  |  |  |  |  |
|  |  |  |  | 706.1892 |  |  |  |  |  |  |
|  |  |  |  | 299.4066 |  |  |  |  |  |  |
|  |  |  |  | 42980.27 |  |  |  |  |  |  |
|  |  |  |  | 36914.26 |  |  |  |  |  |  |
|  |  |  |  | 12560.95 |  |  |  |  |  |  |
