# Supplementary material for: Analysis of plasma metabolic profile, characteristics and enzymes in the progression from chronic hepatitis B to hepatocellular carcinoma
Source: Aging (Albany NY). 2020 Jul 23;12(14):14949–65. doi: 10.18632/aging.103554 (PMC7425494; doi:10.18632/aging.103554)
Supplement: Supplementary Table 2 [file aging-12-103554-s001..docx]

| **ID** | **Uniprot** | **Genename** |
| --- | --- | --- |
| A8K9T9 | A8K9T9 | A8K9T9 |
| MVD | P53602 | MVD |
| RIMKLB | Q9ULI2 | RIMKLB |
| PGP | A6NDG6 | PGP |
| QRSL1 | Q9H0R6 | QRSL1 |
| FPGS | Q05932 | FPGS |
| NANP | Q8TBE9 | NANP |
| GALC | P54803 | GALC |
| UPP1 | Q16831 | UPP1 |
| PAICS | P22234 | PAICS |
| BPGM | P07738 | BPGM |
| ENTPD4 | Q9Y227 | ENTPD4 |
| A8K6K2 | A8K6K2 | A8K6K2 |
| ALLC | Q8N6M5 | ALLC |
| ALPL | B1ANL0 | ALPL |
| YARS2 | Q9Y2Z4 | YARS2 |
| INPP4A | Q96PE3 | INPP4A |
| CPS1 | P31327 | CPS1 |
| DDC | P20711 | DDC |
| TYR | P14679 | TYR |
| OCRL | Q01968 | OCRL |
| SEPHS1 | P49903 | SEPHS1 |
| TAT | P17735 | TAT |
| PNP | P00491 | PNP |
| ACLY | P53396 | ACLY |
| G6PC3 | Q9BUM1 | G6PC3 |
| ATP8B4 | H0YLC1 | ATP8B4 |
| OPLAH | O14841 | OPLAH |
| PAH | P00439 | PAH |
| GLB1 | P16278 | GLB1 |
| MAT1A | Q00266 | MAT1A |
| PNPT1 | Q8TCS8 | PNPT1 |
| SUCLG1 | P53597 | SUCLG1 |
| MIOX | Q9UGB7 | MIOX |
| TYMP | P19971 | TYMP |
| PXDN | Q92626 | PXDN |
| ACP6 | Q9NPH0 | ACP6 |
| INPP5J | Q15735 | INPP5J |
| NUDT5 | Q9UKK9 | NUDT5 |
| MINPP1 | Q9UNW1 | MINPP1 |
| AKR1B10 | O60218 | AKR1B10 |
| FARSLA | Q9Y285 | FARSA |
| LCT | P09848 | LCT |
| NAPRT | Q6XQN6 | NAPRT |
| IL4I1 | Q96RQ9 | IL4I1 |
| ADSS | P30520 | ADSS |
| PRUNE1 | Q5SZF2 | PRUNE1 |
| CCBL1 | Q16773 | KYAT1 |
| NUDT10 | Q9BW91 | NUDT9 |
| NTPCR | Q9BSD7 | NTPCR |
| TPO | P07202 | TPO |
| GAPDHS | O14556 | GAPDHS |
| NSF | P46459 | NSF |
| MTMR2 | Q13614 | MTMR2 |
| NANS | Q9NR45 | NANS |
| TH | P07101 | TH |
| FBP2 | O00757 | FBP2 |
| ABCB4 | P21439 | ABCB4 |
| PDXP | Q96GD0 | PDXP |
| CTPS2 | Q9NRF8 | CTPS2 |
| EPHX2 | P34913 | EPHX2 |
| NUDT16 | Q96DE0 | NUDT16 |
| GALM | Q96C23 | GALM |
| PC | P11498 | PC |
| MTHFS | P49914 | MTHFS |
| DNM3 | Q9UQ16 | DNM3 |
| ACACA | Q13085 | ACACA |
| PNKP | Q96T60 | PNKP |
| LPIN2 | Q92539 | LPIN2 |
| DCTPP1 | Q9H773 | DCTPP1 |
| ENOPH1 | Q9UHY7 | ENOPH1 |
| GOT1 | P17174 | GOT1 |
| ATP1A1 | P05023 | ATP1A1 |
| NAXD | Q8IW45 | NAXD |
| ETNPPL | Q8TBG4 | ETNPPL |
| BCAT1 | P54687 | BCAT1 |
| B4DRW0 | B4DRW0 | B4DRW0 |
| ATP2A1 | O14983 | ATP2A1 |
| SEPSECS | Q9HD40 | SEPSECS |
| CFTR | H0Y8A9 | CFTR |
| PCCA | P05165 | PCCA |
| ENTPD2 | Q9Y5L3 | ENTPD2 |
| INPP1 | P49441 | INPP1 |
| HCG_2005638 | A0A024RCM3 | HCG_2005638 |
| B4E105 | B4E105 | B4E105 |
| IMPAD1 | Q9NX62 | IMPAD1 |
| PYGM | P11217 | PYGM |
| PSPH | P78330 | PSPH |
| GART | P22102 | GART |
| HEL-S-66P | V9HWB5 | HEL-S-66P |
| ASNA1 | O43681 | ASNA1 |
| ACYP1 | P07311 | ACYP1 |
| GDPGP1 | Q6ZNW5 | GDPGP1 |
| PIG59 | A8YXX4 | PIG59 |
| MTAP | P42771 | CDKN2A |
| ACE2 | Q9BYF1 | ACE2 |
| TTL | Q8NG68 | TTL |
| CAD | F8VPD4 | CAD |
| RIMKLA | Q8IXN7 | RIMKLA |
| MCCC2 | Q9HCC0 | MCCC2 |
| ARG2 | P78540 | ARG2 |
| DKFZP666G172 | Q658V6 | DKFZP666G172 |
| IMPA1 | P29218 | IMPA1 |
| ENTPD1 | P49961 | ENTPD1 |
| IARS | P41252 | IARS |
| DHX58 | Q96C10 | DHX58 |
| THTPA | Q9BU02 | THTPA |
| AGMAT | Q9BSE5 | AGMAT |
| PHOSPHO1 | Q8TCT1 | PHOSPHO1 |
| PHYKPL | Q8IUZ5 | PHYKPL |
| GSS | P48637 | GSS |
| TWNK | Q96RR1 | TWNK |
| CDIPT | O14735 | CDIPT |
| PFKFB1 | P16118 | PFKFB1 |
| GCLC | P48506 | GCLC |
| PPP2CA | P67775 | PPP2CA |
| OTC | P00480 | OTC |
| GDE1 | H3BNA5 | GDE1 |
| CARNS1 | A5YM72 | CARNS1 |
| PDP2 | Q9P2J9 | PDP2 |
| PTEN | P60484 | PTEN |
| TMEM55A | E5RIY0 | TMEM55A |
| GLA | P06280 | GLA |
| SPAST | Q9UBP0 | SPAST |
| ALDH18A1 | P54886 | ALDH18A1 |
| MTHFD1L | B7ZM99 | MTHFD1L |
| DPH6 | Q7L8W6 | DPH6 |
| INPP5D | Q92835 | INPP5D |
| DOLPP1 | Q86YN1 | DOLPP1 |
| ACSS2 | Q9NR19 | ACSS2 |
| ACSS3 | Q9H6R3 | ACSS3 |
| SCLY | Q96I15 | SCLY |
| OAT | P04181 | OAT |
| EPRS | P07814 | EPRS |
| GANAB | F5H6X6 | GANAB |
| AARS | P49588 | AARS |
| DKFZP727E011 | Q9NTF0 | DKFZP727E011 |
| B4DXF8 | B4DXF8 | B4DXF8 |
| AGXT | P21549 | AGXT |
| SORD | Q00796 | SORD |
| KYNU | Q16719 | KYNU |
| AGL | P35573 | AGL |
| P4HA1 | P13674 | P4HA1 |
| GATM | P50440 | GATM |
| TREH | O43280 | TREH |
| ADPGK | Q9BRR6 | ADPGK |
| NFS1 | Q9Y697 | NFS1 |
| TARS | P26639 | TARS |
| GCK | P35557 | GCK |
| HK3 | P52790 | HK3 |
| SI | P14410 | SI |
| AGXT2 | Q9BYV1 | AGXT2 |
| LAP3 | P28838 | LAP3 |
| PGLYRP1 | O75594 | PGLYRP1 |
| GANC | H3BN99 | GANC |
| ACY1 | Q03154 | ACY1 |
| PYCR2 | Q96C36 | PYCR2 |
| MOGS | Q13724 | MOGS |
| GPT | P24298 | GPT |
| GBA | P04062 | GBA |
| LALBA | P00709 | LALBA |
| Q8WW81 | Q8WW81 | Q8WW81 |
| GBA3 | Q9H227 | GBA3 |
| FASN | P49327 | FASN |
| E9KL42 | E9KL42 | E9KL42 |
| ACSL3 | O95573 | ACSL3 |
| PPT2 | Q9UMR5 | PPT2 |
| ACSM1 | Q08AH1 | ACSM1 |
